# Unilateral Adrenalectomy, and the Stable Pentadecapeptide BPC 157 as Therapy in Rats—A Cytoprotection Approach

**DOI:** 10.3390/ph19060873

**Published:** 2026-05-30

**Authors:** Ivan Maria Smoday, Vlasta Vukovic, Katarina Oroz, Hrvoje Vranes, Luka Kalogjera, Ozren Gamulin, Josipa Vlainic, Marija Milavic, Suncana Sikiric, Nora Nikolac Gabaj, Domagoj Marijancevic, Antun Koprivanac, Laura Tomic, Sanja Strbe, Ivan Barisic, Lidija Beketic Oreskovic, Mario Kordic, Ante Tvrdeic, Sven Seiwerth, Predrag Sikiric, Alenka Boban Blagaic, Anita Skrtic

**Affiliations:** 1 Department of Pharmacology, School of Medicine, University of Zagreb, 10000 Zagreb, Croatia; ivansmoday1@gmail.com (I.M.S.);; 2 Department of Physics and Biophysics, School of Medicine, University of Zagreb, 10000 Zagreb, Croatia; 3 Laboratory of Oxidative Stress and Advanced Genomics, Division of Molecular Medicine, Institute Ruder Boskovic, 10000 Zagreb, Croatia; 4 Department of Pathology, School of Medicine, University of Zagreb, 10000 Zagreb, Croatia; 5 Department of Chemistry, University Clinical Hospital Center “Sestre Milosrdnice”, 10000 Zagreb, Croatia

**Keywords:** unilateral adrenalectomy, peripheral and central occlusion/occlusion-like syndrome, BPC 157, vascular recovery effect

## Abstract

**Background**. This rat study reveals a new point: the considerable impact of unilateral adrenalectomy, severe vascular and multiorgan failure, occlusion/occlusion-like syndrome, and the stable gastric pentadecapeptide BPC 157 therapy. Based on the recent Fourier transform infrared (FTIR) spectroscopy vascular disturbance studies, particularly those after unilateral adrenalectomy in rats, the noted cytoprotective vascular recovery effect of the BPC 157 therapy may be useful. **Methods**. In rats, unilateral adrenalectomy (at 15 min, 5 h, 24 h) leads to integrated gross and morphological changes, vascular alterations, oxidative stress parameters, molecular markers and occlusion/occlusion-like syndrome and BPC 157 as useful therapy (/kg ig) (10 µg, 10 ng). **Results**. Peripherally and centrally, counteraction includes the lesions (adrenal, brain, heart, lung, liver, kidney, gastrointestinal tract), organ hemorrhage, and thrombosis. Attenuated/eliminated were arrhythmias, intracranial (superior sagittal sinus), portal, caval hypertension, and aortic hypotension. Significant resolution occurred via activation of collateral pathways, the azygos vein (direct blood flow delivery), and the recovered peduncle of the inferior suprarenal artery and superior suprarenal vein. Virchow’s triad circumstances were reversed. Occlusion/occlusion-like syndrome was counteracted as a whole. Also, BPC 157 counteracted adrenal lesions (lipid depletion, congestion). There were higher cortisol values, but still very low, and a shift toward the left of the adrenal compensatory weight increase. For the indicative conclusion along with previous studies, mechanistically, BPC 157 therapy exhibits the NO-system modulation/oxidative stress balance, increases NO-level, counteracts oxidative stress (malondialdehyde (MDA)), upregulates NOS1–3, and VEGF-A expression. **Conclusions**. These effects of BPC 157 therapy and its easy applicability deserve further consideration.

## 1. Introduction

This rat study focused on the considerable impact of unilateral adrenalectomy [1,2,3] and the stable gastric pentadecapeptide BPC 157 therapy [4,5,6]. This may be the very early post-operative intervals, which since the funding studies [7,8,9,10,11,12] have been only sparsely investigated [13,14]. Recent Fourier transform infrared (FTIR) spectroscopy vascular disturbance studies, particularly those after unilateral adrenalectomy in rats, indicate the cytoprotective vascular recovery effect of the stable gastric pentadecapeptide BPC 157 [15,16]. Namely, in a very early post-adrenalectomy period (at 15 min, 5 h, 24 h), FTIR has revealed in the rat aorta extracellular matrix (ECM) degradation, lipid peroxidation, and loss of glycosaminoglycan (GAG) content, leading to impaired vessel viscoelasticity and barrier function [16]. BPC 157 mitigated all these severely disturbed molecular signatures, adrenalectomy-induced vascular injury as a whole [16]. Therefore, from that viewpoint, this may indicate an additional systemic harmful effect, unilateral adrenalectomy → generalized occlusion/occlusion-like syndrome. Likewise, this may indicate a corresponding pleiotropic beneficial effect of BPC 157 therapy in rats after unilateral adrenalectomy.

With respect to BPC 157 therapy, this study further attempts to accommodate this essentially novel point in this very early post-adrenalectomy period, a general failure, and the unilateral adrenalectomy as a severe vascular and multiorgan failure. Currently, this is known to occur after major injuries, major vessel occlusion [17,18,19,20], noxious procedures [21,22,23,24], or various agents’ application [25,26,27,28,29,30], as an occlusion/occlusion-like syndrome [17,18,19,20,21,22,23,24,25,26,27,28,29,30]. That could likely occur amid surgical removal of the adrenal gland. On the other hand, commonly, occlusion/occlusion-like syndrome can be recovered by BPC 157 therapy [17,18,19,20,21,22,23,24,25,26,27,28,29,30]. There, the vascular recovery effect appears by activation of collateral rescuing pathways (i.e., azygos vein direct blood flow delivery) [17,18,19,20,21,22,23,24,25,26,27,28,29,30].

Notably, since its introduction, as noted with recovery of occlusion/occlusion-like syndrome [17,18,19,20,21,22,23,24,25,26,27,28,29,30], stable gastric pentadecapeptide BPC 157 follows the cytoprotection concept, which, formed in the stomach (epithelial and endothelial cell protection, tissue integrity maintenance and recovery) is not organ-specific, but systemic protection (cytoprotection → organoprotection) [31,32,33,34,35,36,37,38,39,40,41,42]. Vice versa, there is a general harmful effect, failed cytoprotection → pleiotropic lesions development [31]. There, BPC 157, as native and stable in human gastric juice, is suggested to act as a cytoprotection mediator, which may be a possible conceptual theory implementation (via cytoprotection agent pleiotropic beneficial effects) to practically resolve various therapy issues [4,5,6]. Therefore, it is easy to apply, including via the per-oral route [4,5,6]. As a continuation of this concept, relevant also for adrenalectomized rats [16] and occlusion/occlusion-like syndromes [17,18,19,20,21,22,23,24,25,26,27,28,29,30], studies on BPC 157 consistently demonstrate, in addition to gastrointestinal protection, its particular cardioprotection, antithrombotic, antiarrhythmic, musculoskeletal, and vascular recovery effects, particularly in ischemia/reperfusion injury [4,5,6]. There, bidirectional regulation (as specifically reviewed [4,5,6]), produced by BPC 157, explains how cytoprotection functions as a unified therapeutic principle [4,5,6]. However, for BPC 157 therapy [4,5,6], human data are limited. It was effectively used in ulcerative colitis trials (phase II) without adverse effects, and later in small studies, also without adverse effects, in knee pain and interstitial cystitis therapy [43,44,45,46]. On the other hand, in toxicology studies, for full details see, i.e., [4], BPC 157 exhibited a harmless limit test, 2 g/kg i.v. or i.g., without adverse effects in mice, and a lethal dose (LD1) was not achieved [4,5,6], high safety confirmed in other studies as well [47,48,49,50,51]. In addition, a very recent study claimed a functional evidence in human arterial tissue [52].

As mentioned, providing the focus on the early course, considerable molecular disturbances occur rapidly in FTIR vascular disturbance studies, particularly in those after unilateral adrenalectomy [15,16]. Within 15 min of peptide administration, spectra already show enhanced amide I and II bands and collagen- and GAG-related features, suggesting early ECM-related remodeling at a molecular level [16]. These changes persist or re-emerge at 24 h, suggesting both an immediate and a sustained phase of matrix adaptation [16].

The full significance of the molecular changes indicated in the aorta vascular wall after unilateral adrenalectomy [16], and the significance of the therapy effect obtained, remains to be further established. This goes to the remaining adrenal gland lesions and recovery, and systemic severe disturbance and its recovery [16]. This could reflect the at least partial substitution of the function of the removed adrenal gland. Likewise, it could reflect the recovery of the remaining adrenal gland’s function, which would otherwise remain dysfunctional for a considerable period [1,2]. Furthermore, taking the adrenal gland in the cytoprotection concept, healing to restore tissue integrity conceptually, this might be a general cytoprotection failure given the essential role of the adrenal gland (vascular fragility) in cytoprotection [3]. Thus, it is likely that in the adrenalectomy model, BPC 157, by activating this protective program within the vascular wall, may have a restorative role [16]. It would enable the circulation and counteract the abrupt hemodynamic and harmful perturbation caused by the removal of one adrenal gland.

Thus, in a very early period (at 15 min, 5 h, 24 h) noted in FTIR studies [16], the rats after unilateral adrenalectomy were investigated for an occlusion/occlusion-like syndrome, i.e., severe vascular and multiorgan failure, blood pressure disturbances, arrhythmias, thrombosis, peripherally and centrally, and organ hemorrhage. These perilous events occurred in rats during the major injury induction [17,18,19,20,21,22,23,24,25,26,27,28,29,30]. Notably, as mentioned, BPC 157 therapy counteracts occlusion/occlusion-like syndromes, as a shared effect in rats with permanent major vessel occlusions, peripheral and central [17,18,19,20], or similar procedures [21,22,23,24] and agents’ [25,26,27,28,29,30] application. These also included an assessment of the cortisol serum level. Likewise, given the general NO-significance [4,53,54,55] and BPC 157 close interaction with many molecular pathways [56,57,58,59,60,61,62,63,64,65,66], particularly with the NO system [58,59,60,67,68,69,70], and acting as a free radical scavenger, the assessment included NO and MDA-tissue levels [4,56,57], and presentation of NOS-1, NOS-2, NOS-3, and VEGF-A gene expression [4] in the adrenal gland. Therefore, this study aimed to link adrenalectomy → adrenal remnant dysfunction → generalized vascular occlusion-like syndrome. Therapy BPC 157 (10 µg or 10 ng/kg) was an early intragastric regimen at 5 min upon adrenalectomy.

## 2. Results

### 2.1. Unilateral Adrenalectomy—General

The focus was on the course after the unilateral adrenalectomy (adrenalectomy → adrenal remnant dysfunction → generalized vascular occlusion-like syndrome). Likely, these indicate cause-consequence relations between the adrenalectomy and the multitude of lesions. There were lesions in the adrenal, brain, heart, lung, liver, kidney, and gastrointestinal tract, progressing thrombosis, arrhythmias, vessel failure, blood pressure disturbances, vs. adrenal gland reactions, and vs. BPC 157 therapy through vascular rescue. Thus, as a particular network, the evidence summarized the particular occlusion/occlusion-like syndrome and the therapy effect. Thereby, the more innate malfunction of the remaining gland, the more severe occlusion/occlusion-like syndrome, this therapy application (one-time applications) can provide an essential, rapid effect that serves as a general defensive response. To this point, the search for the NO-values and MDA values in tissues (i.e., brain, stomach, and adrenal), and NOS-1, NOS-2, NOS-3, and VEGF-A gene expression in adrenals, and revealing direct effect can be important.

#### 2.1.1. Cortisol Assessment

The cortisol values were consistently low in all of the adrenalectomized rats. However, the lowest cortisol serum values (nmol/L) were noted in the controls (19.9 ± 3.4 (15 min), 25.5 ± 5.4 (5 h), and 20.8 ± 7.4 (24 h)). BPC 157-treated rats presented markedly higher values but still far below those in normal rats, 49.9 ± 5.4 (µg), 44.9 ± 4.1 (ng) (15 min), 59.9 ± 5.1 (µg), 50.9 ± 6.1 (ng) (5 h), and 47.8 ± 4.4 (µg), 56.9 ± 4.1 (ng) (24 h) (*p* < 0.05 vs. control at least). Thus, BPC 157 may recover adrenal gland function, at least partly.

#### 2.1.2. Adrenal Gland Weight

Compared to the weight (mg/100g) of the removed left adrenal gland 28 ± 2, the adrenalectomized rats regularly presented a comparative increase in the left-right adrenal in controls (35 ± 2 (15 min), 38 ± 3 (5 h) and 45 ± 3 (24h)) (*p* < 0.05). This increase seems to be advanced in the earliest period in BPC 157 treated rats (45 ± 2 (µg) 43 ± 2 (ng) (15 min)) (*p* < 0.05 vs. control at least), at 5 h period within control values 39 ± 3 (µg) 40 ± 3 (ng)), and at 24 h below control values (36 ± 2 (µg) and 37 ± 2 (ng)) (*p* < 0.05 vs. control at least). Thus, BPC 157 may recover adrenal gland function, shifting toward the left the comparative increase in the left-right adrenal.

#### 2.1.3. Blood Pressure

**Figure 1:**
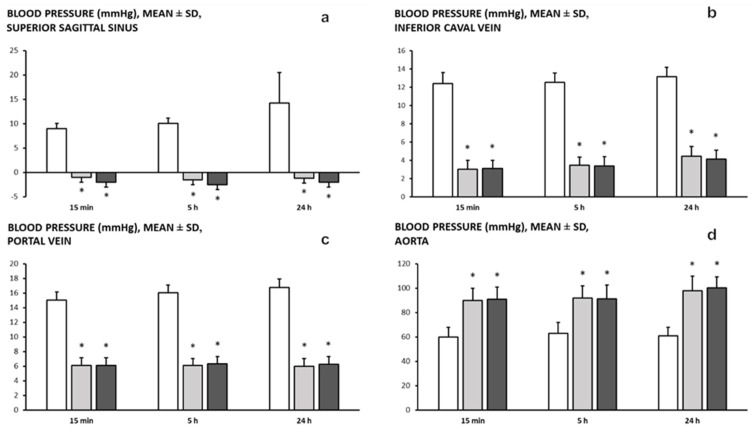
Unilateral adrenalectomy in rats, blood pressure (means ± SD, mmHg) disturbances, intracranial (superior sagittal sinus) (**a**), caval (**b**) and portal (**c**) hypertension and aortal hypotension (**d**) in rats at 15 min, 5 h, and 24 h after surgery, presented in control adrenalectomized rats (white bars), and corrected in BPC 157 treated rats (10 µg/kg, (light gray bars), 10 ng/kg (dark gray bars). * *p* < 0.05 vs. control, at least.

After the adrenalectomy, the adrenalectomized rats presented intracranial (superior sagittal sinus), portal, and caval hypertension, and aortal hypotension, unless they received BPC 157 therapy (Figure 1). In BPC 157 rats, intracranial (superior sagittal sinus), portal, and caval hypertension, and aortal hypotension were attenuated/eliminated.

#### 2.1.4. Thrombosis

**Figure 2:**
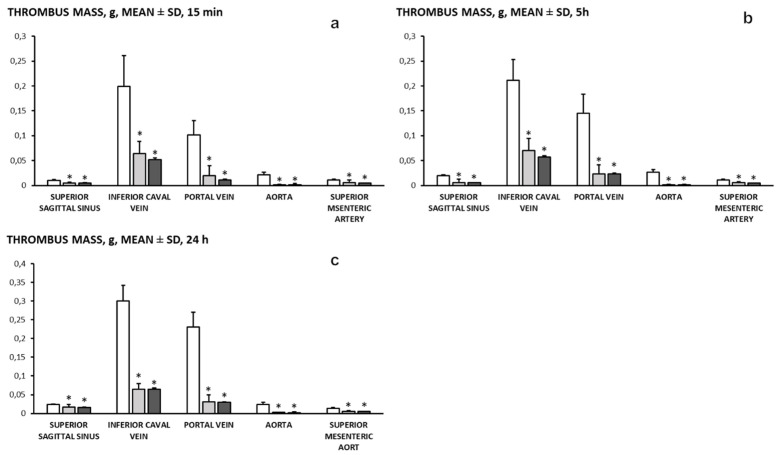
Unilateral adrenalectomy in rats, thrombus mass (mean ± SD, g) in superior sagittal sinus, inferior caval vein, portal vein, abdominal aorta and superior mesenteric artery, at 15 min (**a**), 5 h (**b**), and 24 h (**c**) after surgery. It was consistently presented in control adrenalectomized rats (white bars), and counteracted in BPC 157 treated rats (10 µg/kg (light gray bars), 10 ng/kg (dark gray bars)). * *p* < 0.05 vs. control, at least.

After the adrenalectomy, the adrenalectomized rats presented marked venous and arterial thrombosis, peripherally and centrally, unless they received BPC 157 therapy. BPC 157 therapy almost annihilated thrombosis (Figure 2).

#### 2.1.5. Heart

After the adrenalectomy, the adrenalectomized rats presented tachycardias (beats/min, means ± SD) (410 ± 10 (15 min), 420 ± 10 (5 h), and 410 ± 10 (24 h)), and shortened QTc interval (msec, means ± SD) (137 ± 10 (15 min), 167 ± 5 (5 h), and 170 ± 5 (24 h)). Contrarily, after BPC 157 therapy, there was a counteraction of tachycardias (350 ± 10 (15 min), 330 ± 10 (5 h), and 360 ± 10 (24 h) (BPC 157 μg); 360 ± 10 (15 min), 340 ± 10 (5 h), and 330 ± 10 (24 h) (BPC 157 ng)) (*p* < 0.05 vs. control at least). Likewise, there was a counteraction of shortened QTc interval (195 ± 5 (15 min), 187 ± 5 (5 h), and 185 ± 5 (24 h) (BPC 157 μg); 190 ± 5 (15 min), 185 ± 5 (5h), and 195 ± 5 (24 h) (BPC 157 ng)) (*p* < 0.05 vs. control at least). Thus, BPC 157 may recover heart function in adrenalectomized rats.

#### 2.1.6. Brain, Blood Vessel, and Heart Volume

**Figure 3:**
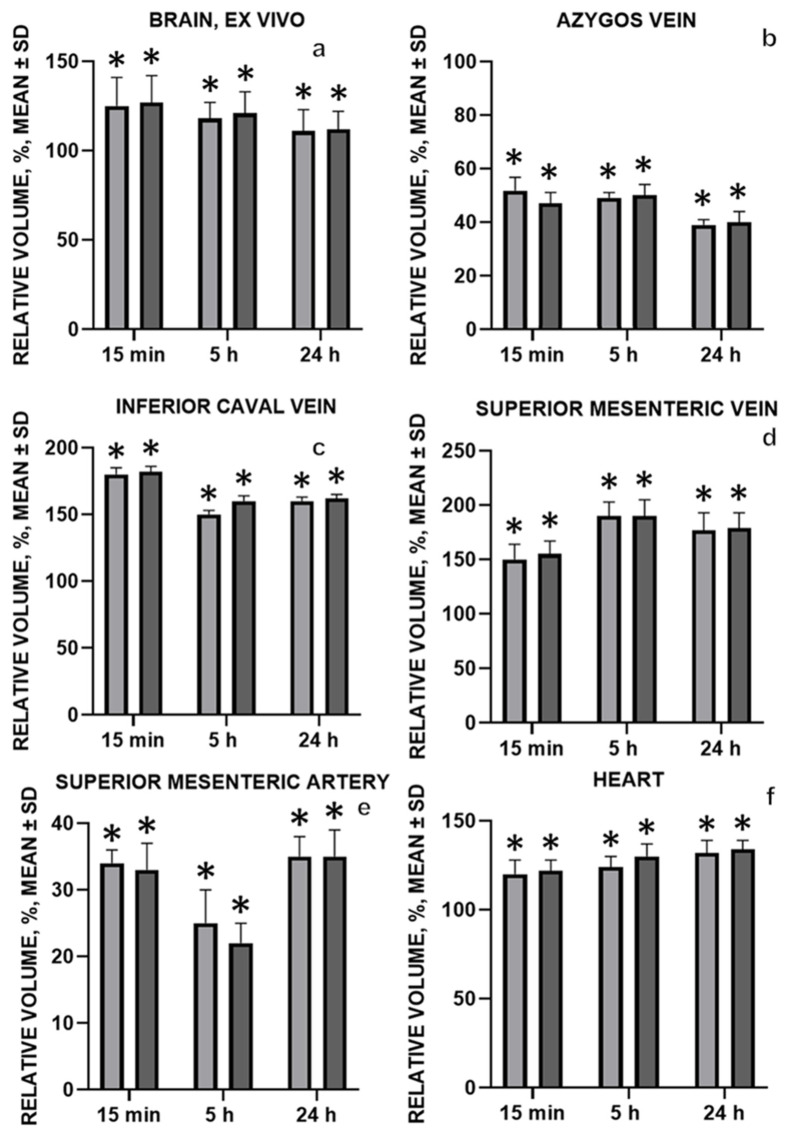
Unilateral adrenalectomy in rats, relative volume (brain (**a**), azygos vein (**b**), inferior caval vein (**c**), superior mesenteric vein (**d**), superior mesenteric artery (**e**), and heart (**f**), %, control/treated, assessed at 15 min, 5 h, and 24 h after surgery), presented in BPC 157 treated rats (mean ± SD, 10 µg/kg (light gray bars), 10 ng/kg (dark gray bars). * *p* < 0.05 vs. control, at least.

**Figure 4:**
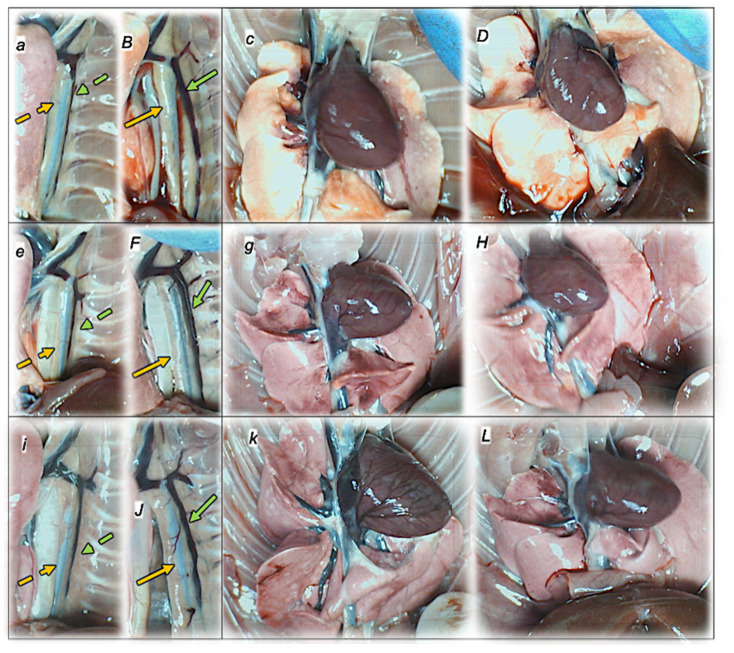
Azygos vein, aorta and heart gross presentation. Unilateral adrenalectomy in rats, immediate course, and course thereafter as an occlusion/occlusion-like syndrome in control rats (small italic letters, dashed arrows) (**a**,**c**,**e**,**g**,**i**,**k**) and BPC 157-treated rats (capital italic letters, full arrows) (**B**,**D**,**F**,**H**,**J**,**L**). Azygos vein (green arrows) and aorta (yellow arrows) (**a**,**B**,**e**,**F**,**i**,**J**). Heart (**c**,**D**,**g**,**H**,**k**,**L**). Control. Considerable failure. Collapsed azygos vein and aorta (**a**,**e**,**i**). Dilated heart (**c**,**g**,**k**). BPC 157 therapy. Recovery. Rescued azygos vein and aorta (**B**,**F**,**J**). Rescued heart, (**D**,**H**,**L**). Adrenalectomy-times: 15 min (**a**,**B**,**c**,**D**) (i), 5 h (**e**,**F**,**g**,**H**) (ii) or 24 h (iii) (**i**,**J**,**k**,**L**). Medication (ig) immediately after unilateral adrenalectomy, saline (controls) or BPC 157.

**Figure 5:**
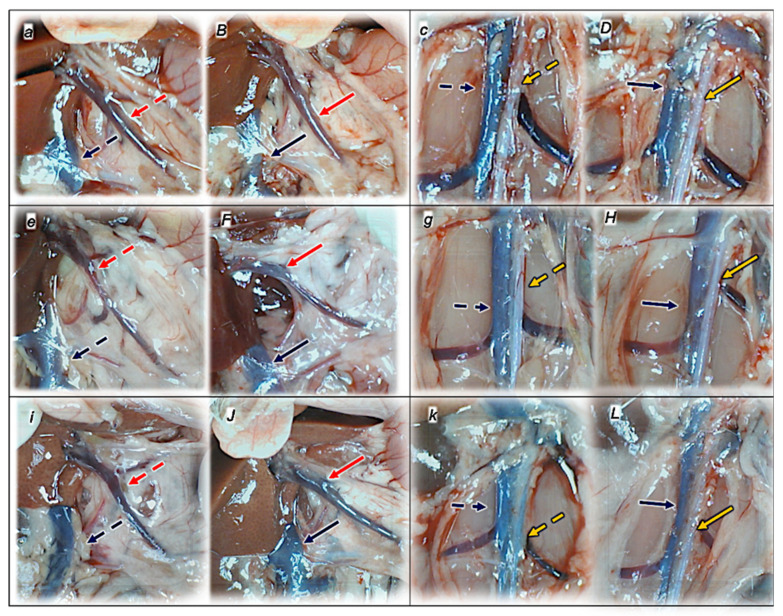
Blood vessels’ gross presentation. Unilateral adrenalectomy in rats, immediate course, and course thereafter as an occlusion/occlusion-like syndrome in control rats (small italic letters, dashed arrows) (**a**,**c**,**e**,**g**,**i**,**k**) and BPC 157-treated rats (capital italic letters, full arrows) (**B**,**D**,**F**,**H**,**J**,**L**). Superior mesenteric vein (red arrows) and inferior caval vein (blue arrows) (**a**,**B**,**e**,**F**,**i**,**J**). Inferior caval vein (blue arrows) and abdominal aorta (yellow arrows) (**c**,**D**,**g**,**H**,**k**,**L**). Control. Considerable vascular failure. Congested superior mesenteric vein and inferior caval vein (**a**,**e**,**i**). Congested inferior caval vein and collapsed aorta (**c**,**g**,**k**). BPC 157 therapy. Vascular recovery. Rescued superior mesenteric vein and inferior caval vein (**B**,**F**,**J**). Rescued inferior caval vein and abdominal aorta (**D**,**H**,**L**). Adrenalectomy-times: 15 min (**a**,**B**,**c**,**D**) (i), 5 h (**e**,**F**,**g**,**H**) (ii) or 24 h (iii) (**i**,**J**,**k**,**L**). Medication (ig) immediately after unilateral adrenalectomy, saline (controls) or BPC 157.

**Figure 6:**
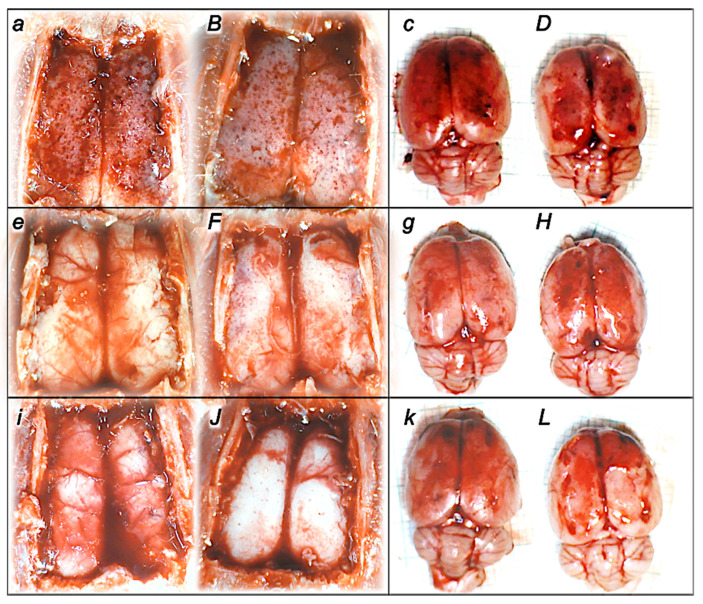
Brain, gross presentation. Unilateral adrenalectomy in rats, immediate course, and course thereafter as an occlusion/occlusion-like syndrome in control rats (small italic letters) (**a**,**c**,**e**,**g**,**i**,**k)** and BPC 157-treated rats (capital italic letters) (**B**,**D**,**F**,**H**,**J**,**L**). Brain (in vivo) (**a**,**B**,**e**,**F**,**i**,**J**). Brain (ex vivo) (**c**,**D**,**g**,**H**,**k**,**L**). Control. Brain swelling. In vivo (**a**,**e**,**i**). Ex vivo (**c**,**g**,**k**). BPC 157 therapy. Recovery. Counteracted brain swelling. In vivo (**B**,**F**,**J**). Ex vivo (**D**,**H**,**L**). Adrenalectomy-times: 15 min (**a**,**B**,**c**,**D**) (i), 5 h (**e**,**F**,**g**,**H**) (ii) or 24 h (iii) (**i**,**J**,**k**,**L**). Medication (ig) immediately after unilateral adrenalectomy, saline (controls) or BPC 157.

**Figure 7:**
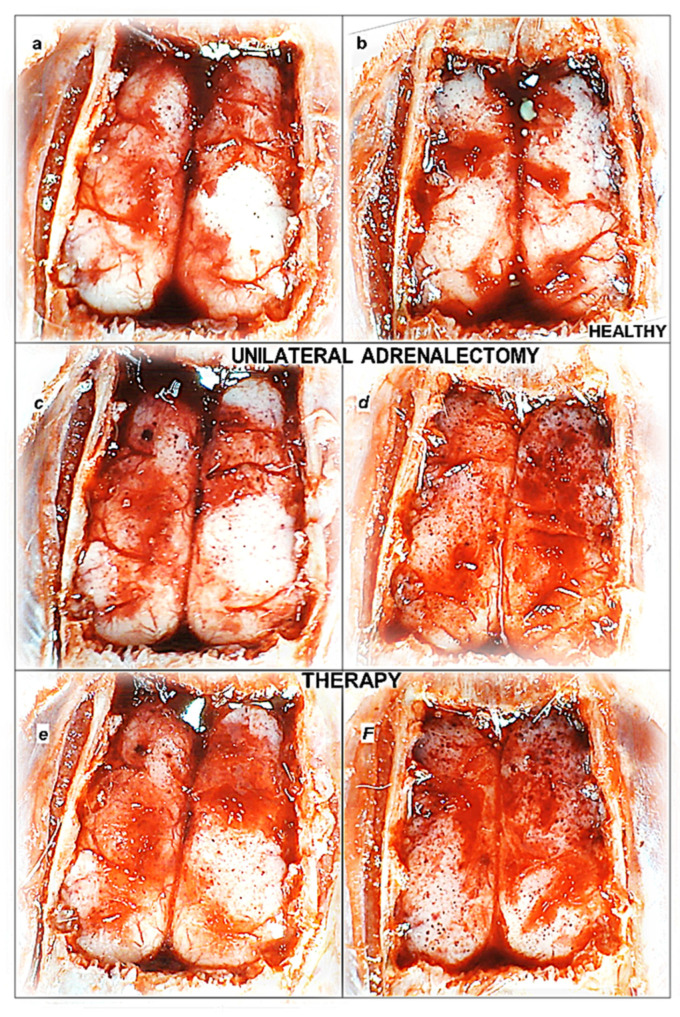
Brain, gross presentation, healthy, adrenalectomy, therapy. Unilateral adrenalectomy in rats, immediate course, as an initiated occlusion/occlusion-like syndrome. Illustrative presentation of brain, in healthy rats (normal small letters) (**a**,**b**), immediately after unilateral adrenalectomy (small italic letters) (**c**,**d**), and immediately after medication (**e**,**F**) in control rats (small italic letters) (**e**) and BPC 157-treated rats (capital italic letters) (**F**). Considerable failure (brain swelling (**c**–**e**), or recovery (counteracted brain swelling (**F**)) occurred in adrenalectomized rats depending on upon receiving (ig) saline (controls) (**e**) or BPC 157 (**F**) immediately after unilateral adrenalectomy. Note, unilateral adrenalectomy produces an immediate brain volume increase (relative to healthy values (100%), %, means ± SD, adrenalectomy/healthy) (**c**,**d**) of 115 ± 2 (*p* < 0.05, at least). Furthermore, application of saline, given intragastrically, produced considerable deterioration, an additional swelling increase (relative to adrenalectomy values (100%), %, means ± SD, saline/adrenalectomy) 111 ± 1 (*p* < 0.05, at least). Contrarily, after adrenalectomy, there is an opposite recovering course in BPC 157-treated rats. Application of BPC 157, given intragastrically, produced an immediate swelling decrease (relative to adrenalectomy values (100%), %, means ± SD, BPC 157/adrenalectomy) 88 ± 2 (µg), 85 ± 2 (ng) (*p* < 0.05, at least). Therefore, with respect to regular control course in adrenalectomized rats, a particular outcome (brain volume decreased) immediately after BPC 157 therapy application (relative to control (saline) values (**e**) (100%), %, means ± SD, %, control/treated) (**F**) 125 ± 2 (µg), 123 ± 2 (ng).

**Figure 8:**
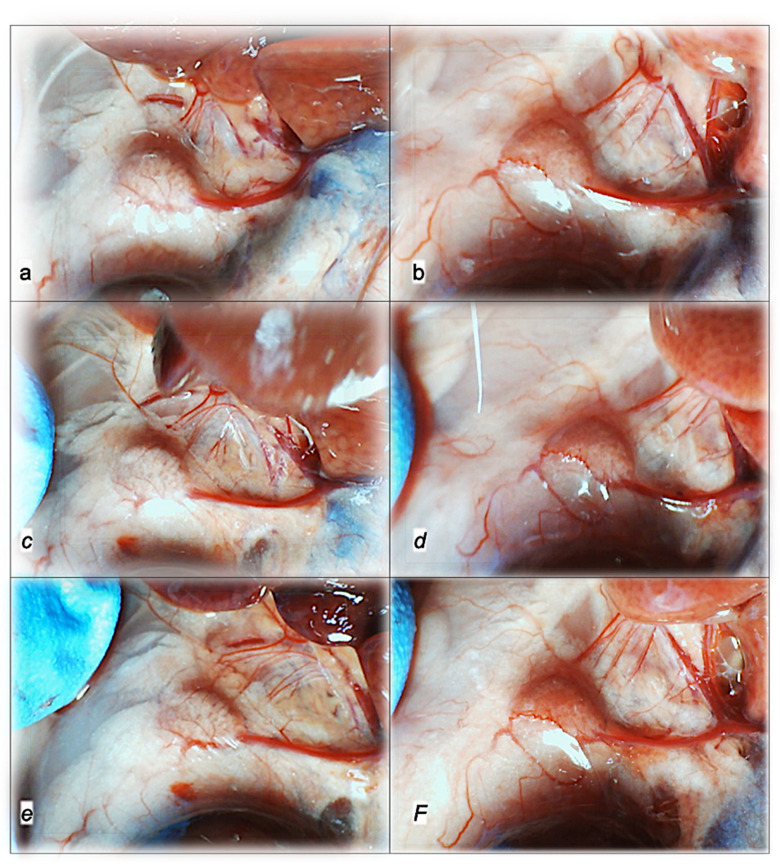
Right adrenal gland vessel gross presentation, healthy, unilateral adrenalectomy, therapy. Healthy rats before unilateral adrenalectomy (normal small letters, (**a**,**b**)). Adrenalectomy (italic letters). Rats after unilateral adrenalectomy, an initiation of occlusion/occlusion-like syndrome, before medication (**c**,**d**). Adrenalectomy + medication. Rats after unilateral adrenalectomy, immediately after ig saline medication (controls, small italic letter) (**e**) or BPC 157 medication (capital italic letters) (**F**). Considerable failure (collapsed and congested vessel) (unilateral adrenalectomy, (**c**–**e**)). Recovery (rescued vessel, BPC 157 therapy) (**F**). Presentation in adrenalectomized rats immediately upon mediation at 2 min adrenalectomy-times, depending on receiving (ig) saline (controls) (**e**) or BPC 157 (**F**) after unilateral adrenalectomy. Note, given vessel in normal rats (normal small letters, (**a**,**b**)), unilateral adrenalectomy produces an immediate peduncle of the inferior suprarenal artery and superior suprarenal vein volume decrease (relative to healthy values (100%), %, means ± SD, *p* < 0.05, at least) (**c**,**d**) of 88 ± 2. With medication, there is a particular distinctive outcome. Either peduncle volume further decreased immediately after saline (ig) (74 ± 2) (**e**), or after BPC 157 therapy application, a complete reversal occurred (peduncle volume further increased) (**F**) (114 ± 2 (µg), 116 ± 3 (ng)) (relative to control (saline), *p* < 0.05, at least).

**Figure 9:**
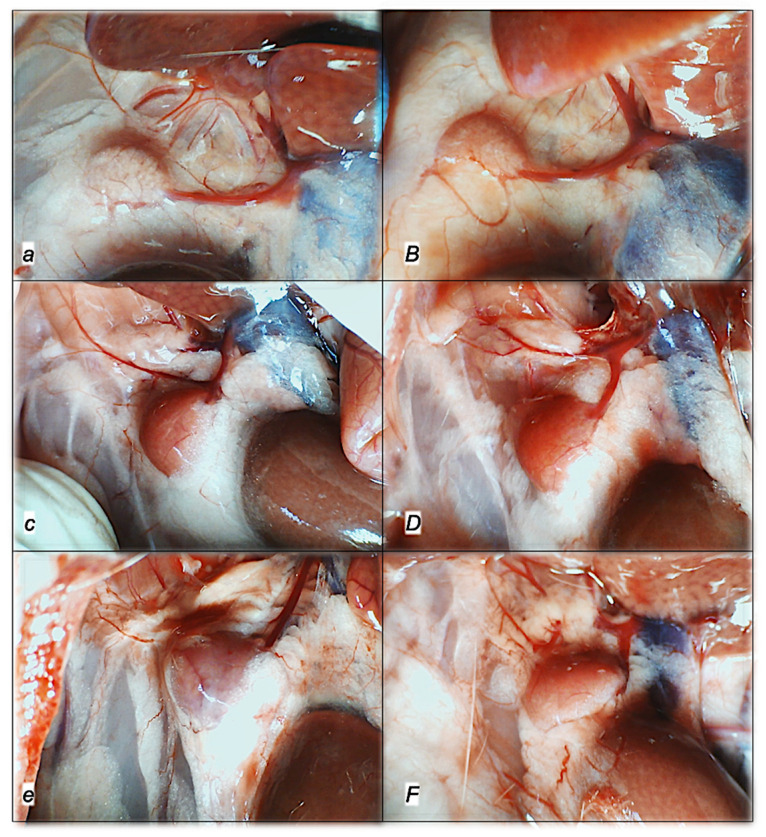
Right adrenal gland vessel gross presentation. Unilateral adrenalectomy in rats, immediate course, and course thereafter as an occlusion/occlusion-like syndrome in control rats (small italic letters) (**a**,**c**,**e**) and BPC 157 treated rats (capital italic letters) (**B**,**D**,**F**). Control. Considerable failure. Collapsed and congested vessel (**a**,**c**,**e**). BPC 157 therapy. Recovery. Rescued vessel (**B**,**D**,**F**). Adrenalectomy-times: 15 min (**a**,**B**) (i), 5 h (**c**,**D**) (ii) or 24 h (iii) (**e**,**F**). Medication (ig) immediately after unilateral adrenalectomy, saline (controls) or BPC 157. As seen with peduncle in the immediate course (Figure 8), in the further course, this distinction (volume decrease (saline) vs. volume increase (BPC 157), consistently remained. 86 ± 2 at 15 min, 64 ± 2 at 5 h, 76 ± 2 at 24 h (saline) vs. 145 ± 2 (µg), 140 ± 3 (ng) at 15 min, 105 ± 2 (µg), 103 ± 2 (ng) at 5 h, 110 ± 3 (µg), 113 ± 2 (ng) at 24 h (BPC 157). (relative to control (saline), *p* < 0.05, at least).

In adrenalectomized rats, as evidenced by gross assessment, marked brain swelling, congested inferior caval vein, and superior mesenteric vein, collapsed abdominal aorta, dilated heart and collapsed azygos vein, and collapsed peduncle instantly occurred upon surgery (Figure 3, Figure 4, Figure 5, Figure 6, Figure 7, Figure 8 and Figure 9). This is consistent with intracranial (superior sagittal sinus), portal and caval hypertension, and aortal hypotension. Together, these findings fully accord with the volume increase in the brain, blood vessels (inferior caval vein and superior mesenteric vein), and dilated heart, and the reduced volume of collapsed vessels (azygos vein, abdominal aorta, peduncle). As mentioned, the described gross presentation of malfunctioning and blood pressure disturbances (i.e., intracranial (superior sagittal sinus), portal and caval hypertension, and aortal hypotension) were fully eliminated/attenuated by BPC 157 therapy. Likewise, the volume increase in the brain, blood vessels (inferior caval vein and superior mesenteric vein), and dilated heart, and the reduced volume of collapsed vessels (azygos vein, abdominal aorta, peduncle) were all accordingly reversed by BPC 157 therapy. The reversal appeared rapidly upon BPC 157 therapy application.

### 2.2. Microscopy

To determine the effectiveness of BPC 157 therapy in such acute tissue injury, a mostly semiquantitative assessment, previously carried out in our occlusion/occlusion-like syndrome reports [17,18,19,20,21,22,23,24,25,26,27,28,29,30], was applied to point-out the lesions across multiple organs. Notably, it was not used as a stand-alone proof. Like in previous reports [17,18,19,20,21,22,23,24,25,26,27,28,29,30], such supportive morphological evidence stands along with other evidence. Matching was complete with i.e., assessed gross organ and vessel presentation, ECG recording, determined blood pressure disturbances, intracranial, portal and caval hypertension, and aortal hypotension, thrombosis, assessed peripherally and centrally, and further mechanistic assessment.

#### 2.2.1. Heart

**Figure 10:**
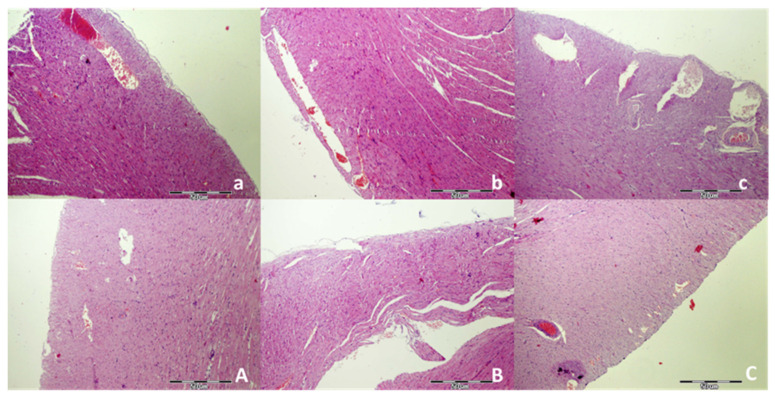
Illustrative microscopic presentation of cardiac congestion (H&E staining; magnification 100×) in control (**a**–**c**) and treated (**A**–**C**) groups of animals at the end of the time intervals following the procedure: 15 min (**a**,**A**), 5 h (**b**,**B**), and 24 h (**c**,**C**). Pronounced myocardial congestion was consistently observed in all control groups (**a**–**c**), showing time-dependent progression, whereas in the treated groups, only mild congestion was found at the 24-h sacrifice point (**C**).

After unilateral adrenalectomy, control rats exhibited pronounced congestion and dilatation of coronary arteries and their intramyocardial branches up to the subendocardial area, progressing during the post-surgery period. Contrarily, no, or only mild congestion, was observed in BPC 157 rats (Table 1, Figure 10).

#### 2.2.2. Lung

**Figure 11:**
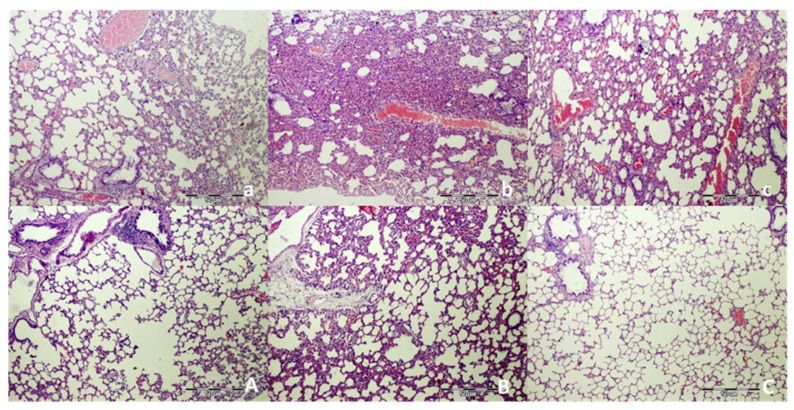
Illustrative microscopic presentation of lung tissue damage (H&E staining; magnification 100×) in control (**a**–**c**) and treated (**A**–**C**) groups of animals at the end of the time intervals following the procedure: 15 min (**a**,**A**), 5 h (**b**,**B**), and 24 h (**c**,**C**). In the control groups, pronounced congestion of the pulmonary parenchyma was consistently observed, with progressive intra-alveolar hemorrhage evident at the 24-h time point (**a**–**c**). In the treated groups (**A**–**C**), only mild congestion of the lung tissue was observed at 24 h after the procedure (**C**).

After unilateral adrenalectomy, the control rats presented thickening of the alveolar membranes due to capillary congestion, pulmonary edema, and dilatation of larger blood vessels focal and intralveolar hemorrhage in all three assessment time periods. BPC 157 treated rats exhibited only mild congestion of the lung tissue (Table 1, Figure 11).

#### 2.2.3. Liver

**Figure 12:**
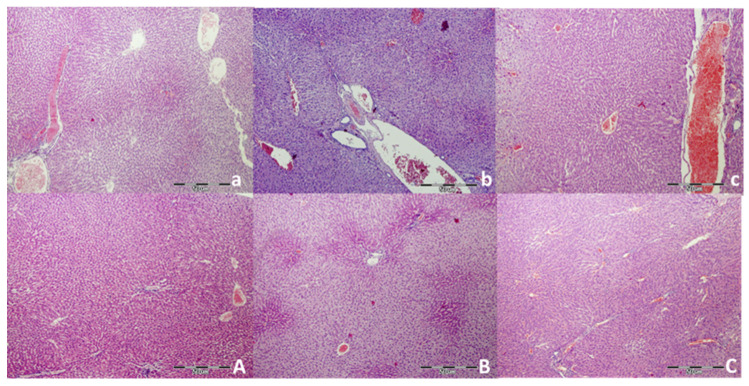
Illustrative microscopic presentation of damaged liver tissue (H&E staining; magnification 100×) in control (**a**–**c**) and treated (**A**–**C**) groups of animals at the end of the time intervals following the procedure: 15 min (**a**,**A**), 5 h (**b**,**B**), and 24 h (**c**,**C**). In the control groups, marked dilatation and congestion of blood vessels in the portal areas, central veins, and sinusoids were observed at 15 min (**a**), 5 h (**b**), and 24 h (**c**) after the procedure. In the treated groups, mild dilatation and congestion of blood vessels were noted, with considerably less dilatation of the portal areas compared to the control groups, particularly at the longest time interval of 24 h (**c**,**C**).

Control rats exhibited marked dilatation and congestion of blood vessels in the portal areas, central veins, and sinusoids in liver tissue in all three assessment time periods. Contrarily, BPC 157 rats exhibited mild dilatation and congestion of blood vessels, with considerably less dilatation of the portal areas compared to the control groups, particularly at the longest time interval of 24 h (Table 1, Figure 12).

#### 2.2.4. Stomach

**Figure 13:**
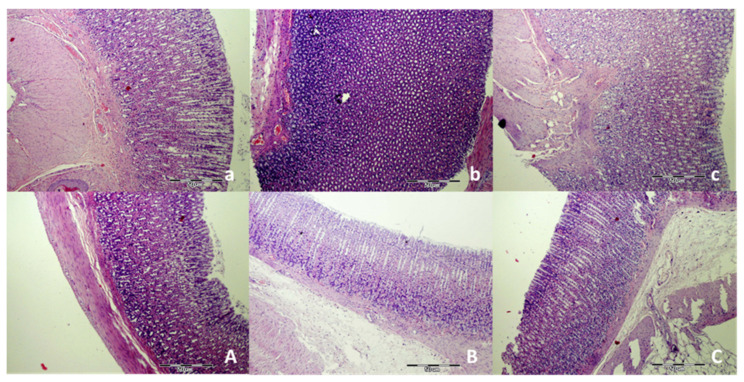
Illustrative microscopic presentation of damaged gastric tissue (H&E staining; magnification 100×) in control (**a**–**c**) and treated (**A**–**C**) groups of animals at the end of the time intervals following the procedure: 15 min (**a**,**A**), 5 h (**b**,**B**), and 24 h (**c**,**C**). In the control groups (**a**–**c**), but not in BPC 157 rats, congestion of the gastric wall was observed, showing progression over time.

In the control groups, predominantly hyperemic and hemorrhagic changes in the gastric wall were observed, which worsened over time. BPC 157 treatment demonstrated protective effects against these changes at all time intervals (Table 1, Figure 13). Notably, grossly, small lesions occurred in control rats, while no lesions could be seen in treated rats.

#### 2.2.5. Small Intestine

**Figure 14:**
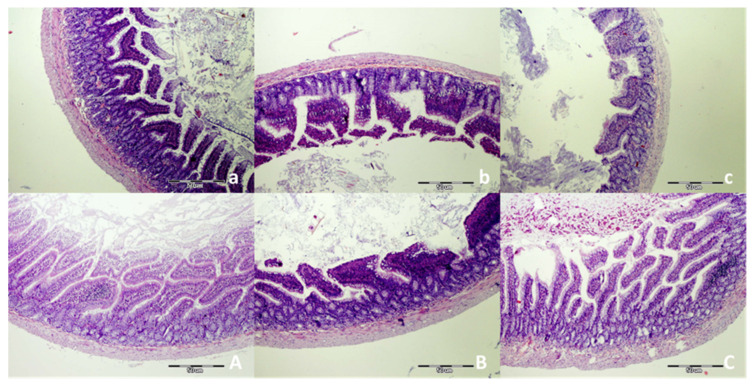
Illustrative microscopic presentation of small intestine damage (H&E staining; magnification 100×) in control (**a**–**c**) and treated (**A**–**C**) groups of animals at the end of the time intervals following the procedure: 15 min (**a**,**A**), 5 h (**b**,**B**), and 24 h (**c**,**C**). In the control groups, pronounced congestion was consistently observed, being more marked at 24 h after the procedure (**c**), whereas animals treated with the pentadecapeptide BPC 157 showed only mild changes at 24 h (**C**).

In the small intestine, marked congestion was observed at all time intervals, with more pronounced changes at 24 h. Animals treated with the pentadecapeptide BPC 157 showed only mild changes at 24 h (Table 1, Figure 14).

#### 2.2.6. Kidney

**Figure 15:**
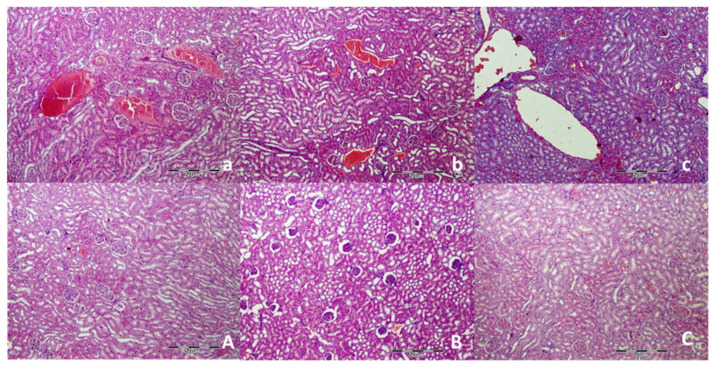
Illustrative microscopic presentation of kidney tissue damage (H&E staining; magnification 100×) in control (**a**–**c**) and treated (**A**–**C**) groups of animals at the end of the time intervals following the procedure: 15 min (**a**,**A**), 5 h (**b**,**B**), and 24 h (**c**,**C**). In the control groups (**a**–**c**), the changes were present at all time intervals, whereas in the BPC 157–treated animals, these changes were counteracted, with only mild dilatation and congestion of blood vessels observed at the 24 h interval (**C**).

After unilateral adrenalectomy, there was marked renal vascular congestion, and interstitial edema and hemorrhage, as well as blood within glomeruli in control rats. Contrarily, in the BPC 157–treated animals these changes were counteracted. BPC 157 rats exhibited only mild dilatation and congestion of blood vessels at the 24 h interval (Table 1, Figure 15).

#### 2.2.7. Adrenal Gland—Zona Glomerulosa

**Figure 16:**
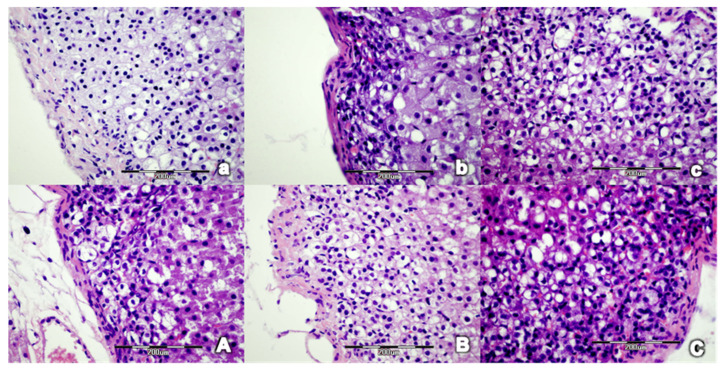
Illustrative microscopic presentation of adrenal gland changes in the zona glomerulosa (H&E staining; magnification 200×) in control (**a**–**c**) and treated (**A**–**C**) groups of animals at the end of the time intervals following the procedure: 15 min (**a**,**A**), 5 h (**b**,**B**), and 24 h (**c**,**C**). In the control animals, a loss of lipid vacuoles was observed. In the BPC 157–treated animals, the loss of lipid vacuoles was reduced compared to the control animals.

The zona glomerulosa is shown with a marked loss of intracellular lipid vacuoles in the control animals. This loss decreased over time in the control animals. Contrarily, in the BPC 157–treated animals the lipid vacuoles were impressively preserved at all three time intervals (Table 1, Figure 16).

#### 2.2.8. Adrenal Gland—Zona Fasciculate

**Figure 17:**
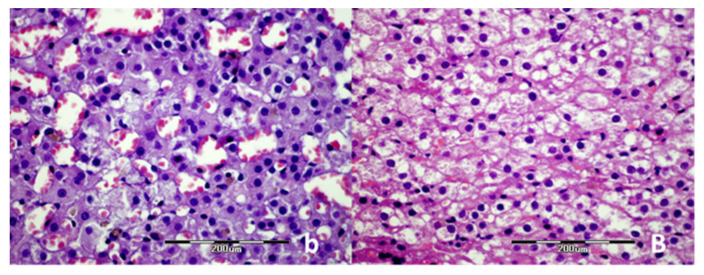
Illustrative microscopic presentation of adrenal gland changes in the zona fasciculata (H&E staining; magnification 200×) in control (**b**) and treated (**B**) groups of animals at the end of the 5 h interval following the procedure. In the control animals, loss of lipid vacuoles and congestive changes were observed. In the BPC 157–treated animals, the loss of lipid vacuoles was reduced compared to the control animals.

In contrast to the zona reticularis, the lipid vacuoles in the zona fasciculate decreased over time. Initially, the vacuoles also decreased in the treated animals. Then, in subsequent intervals, in the BPC 157–treated animals, the lipid vacuoles recovered, and remained preserved (Table 1, Figure 17).

#### 2.2.9. Adrenal Gland—Medulla

**Figure 18:**
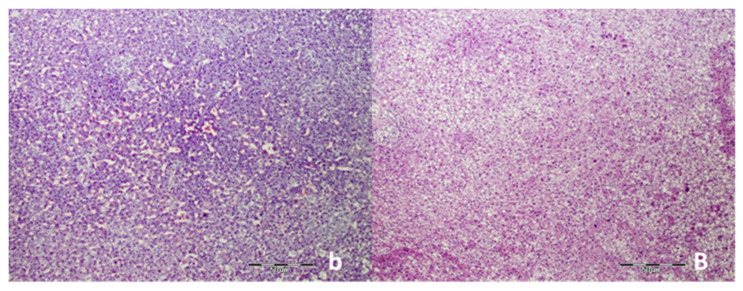
Illustrative microscopic presentation of pronounced congestive changes in the adrenal medulla (H&E staining; magnification 100×) in control (**b**) and physiological hyperemia in treated (**B**) groups of animals at the end of the 5 h interval following the procedure.

Pronounced congestive changes in the adrenal medulla occurred in control rats. This was counteracted in BPC 157 treated rats. They exhibited a presentation close to the physiological hyperemia (Table 1, Figure 18).

#### 2.2.10. Brain

**Figure 19:**
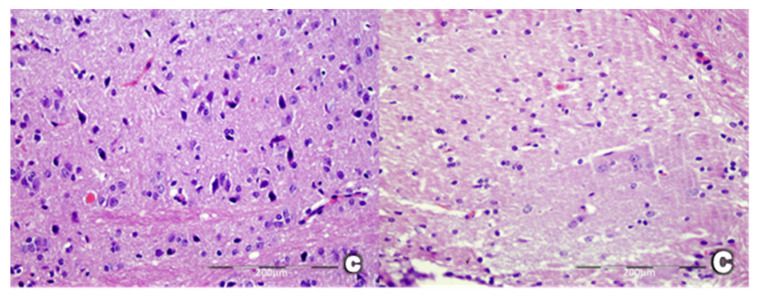
Illustrative microscopic presentation of the hypothalamus (H&E staining; magnification 200×) in control (**c**) and treated (**C**) groups of animals at the end of the 24 h interval following the procedure. Marked neuronal karyopyknosis was observed in the control groups (**c**), whereas only mild karyopyknosis was present in the treated groups (**C**).

**Figure 20:**
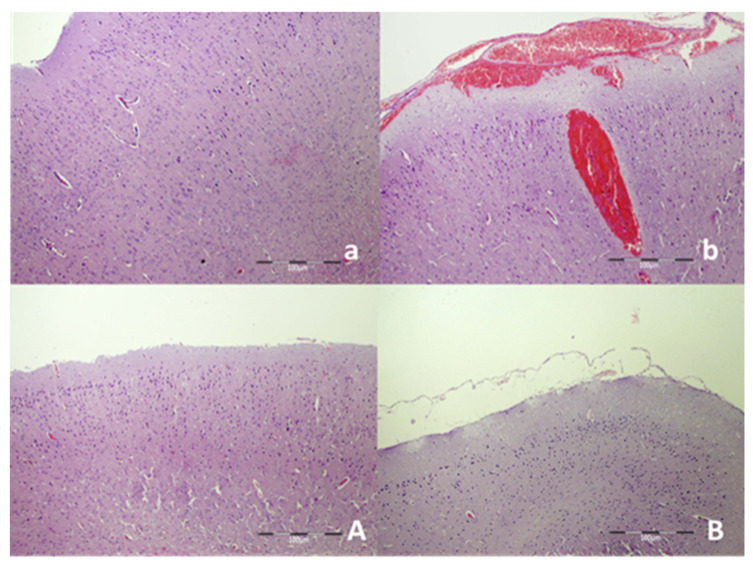
Illustrative microscopic presentation of the cerebral cortex (H&E staining; magnification 100×) in control (**a**,**b**) and treated (**A**,**B**) groups of animals at the end of the time intervals following the procedure: 5 h (**a**,**A**) and 24 h (**b**,**B**). In the control groups (**a**,**b**), marked edema and vascular hyperemia were observed, showing time-dependent progression, whereas in the treated groups only a mild degree of edema and hyperemia was present at 5 h (**A**) and at the 24 h sacrifice interval (**B**). In addition, subarachnoid hemorrhage was demonstrated in the control animal sample at 5 h after the procedure (**a**).

**Figure 21:**
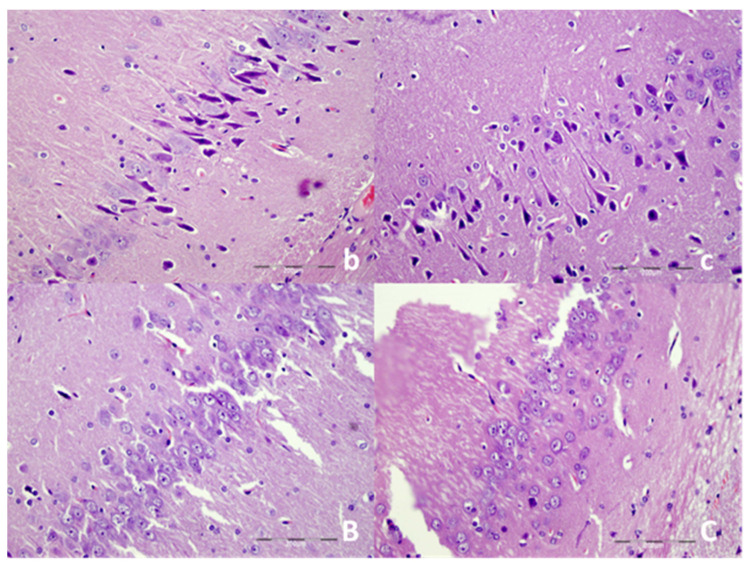
Illustrative microscopic presentation of the hippocampus (H&E staining; magnification 200×) in control (**b**,**c**) and treated (**B**,**C**) groups of animals at the end of the time intervals following the procedure: 5 h (**b**,**B**) and 24 h (**c**,**C**). In the control groups (**b**,**c**), marked neuronal karyopyknosis was observed with time-dependent progression, whereas in the treated groups only a mild degree of karyopyknosis was found at 5 h (**B**) and at the 24 h sacrifice interval (**C**).

Pronounced edema and hyperemia were observed in all analyzed regions of the central nervous system at all three time intervals (15 min, 5 h, and 24 h) in the control groups (Figure 19, Figure 20 and Figure 21, Table 2), and these changes intensified over time, while edema was less pronounced in the BPC 157–treated groups. Congestion was also observed in all analyzed regions of the central nervous system, being most pronounced at the 24 h interval. In contrast, only subtle signs of congestion were visible in the treated groups. Intraventricular hemorrhage affecting the third cerebral ventricle was observed in the control groups at 24 h, but was not present in the BPC 157–treated groups. Control animals also exhibited significant neurodegenerative changes in the central nervous system, including an increased number of karyopyknotic cells, predominantly affecting the hypothalamus and hippocampus, which worsened over time. These changes were mitigated in animals treated with the pentadecapeptide BPC 157.

### 2.3. Oxidative Stress

**Figure 22:**
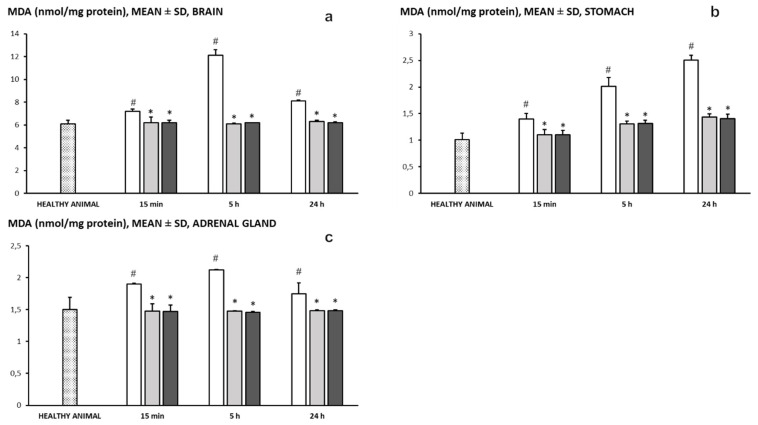
Oxidative stress (MDA, nmol/mg protein, means ± SD), in healthy rats (dotty bars) and in the hemiadrenactomized rats after 15 min, 5 h, and 24 h after surgery presented in brain (**a**), stomach (**b**), and adrenal gland (**c**), increased in control adrenalectomized rats (white bars), and counteracted in BPC 157 treated rats (10 µg/kg, (light gray bars), 10 ng/kg (dark gray bars)). # *p* < 0.05, at least vs. healthy; * *p* < 0.05, at least vs. control.

MDA levels generally increased in adrenalectomized control rats compared with healthy rats. Conversely, MDA levels in BPC 157–treated animals remained unchanged, similar to those in healthy rats. Thus, BPC 157 therapy strongly counteracted the raised MDA levels (Figure 22).

### 2.4. NO Levels

**Figure 23:**
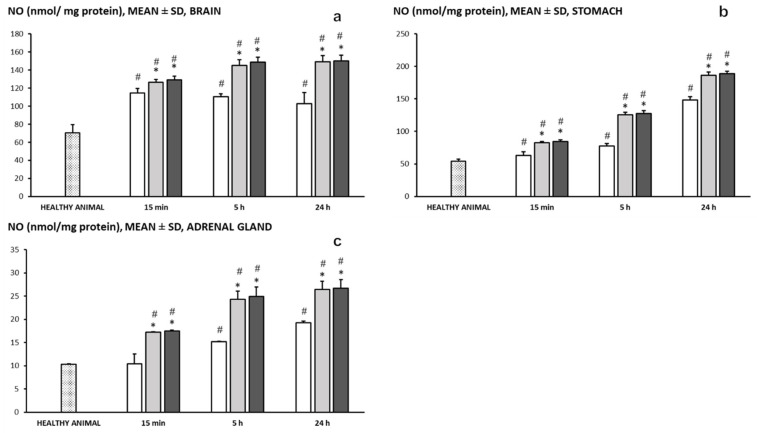
NO levels (nmol/mg protein, means ± SD), in healthy rats (dotty bars) and in the hemiadrenactomized rats after 15 min, 5 h, and 24 h after surgery presented in brain (**a**), stomach (**b**), and adrenal gland (**c**), increased in control adrenalectomized rats (white bars), and even more in BPC 157 treated rats (10 µg/kg, (light gray bars), 10 ng/kg (dark gray bars)). # *p* < 0.05, at least vs. healthy; * *p* < 0.05, at least vs. control.

NO levels showed generally increased values in adrenalectomized rats compared with healthy rats. Given strong beneficial effects and counteraction of the increased MDA levels, it is interesting that NO levels in BPC 157–treated animals were additionally increased compared to the levels in control animals (Figure 23).

### 2.5. Gene Analysis

**Figure 24:**
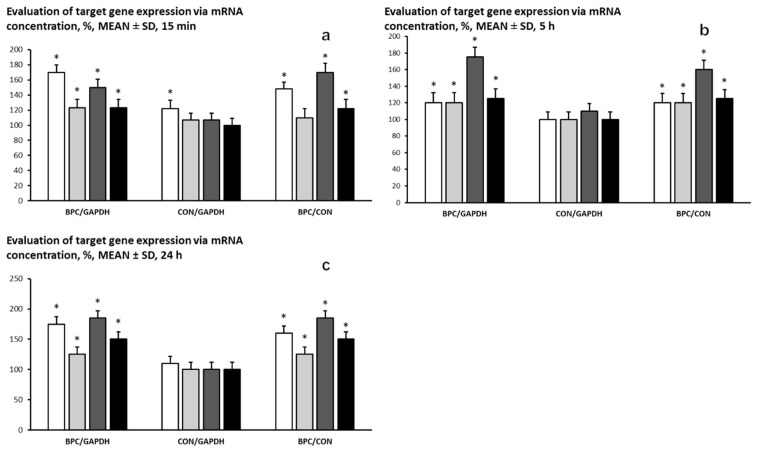
mRNA expression. Real-time PCR determination of mRNA expression of a set of targeted genes in adrenal gland tissue samples, NOS 1 (white bars), NOS 2 (light gray bars), NOS 3 (dark gray bars), and VEGF-A (black bars) time interval, 15 min (**a**), 5 h (**b**), and 24 h (**c**) after surgery. Results are expressed as percentage changes (means ± SD) of BPC 157 10 ng/kg (intragastric application) relative to control samples. *p* < 0.05 is indicated with an asterisk (*). Results without an asterisk have no biological difference from control samples.

Gene analysis showed generally increased expression of NOS 1, NOS 2, NOS 2, and VEGF-A in BPC 157–treated animals compared to the expression in control animals and the housekeeping gene GAPDH, whereas the expression remained largely unchanged in the control groups (Figure 24).

Thus, as a summary after unilateral adrenalectomy, appears Table 3 as a summary of statistical comparisons across systemic, organ-specific, functional, morphometric, and molecular endpoints. There is a full mechanistic hierarchy, structural damage (organs, brain, vessels), functional collapse (blood pressure, heart rate, QTc), morphometric system failure (volume changes), and molecular mechanism layer (MDA (oxidative stress), NO (nitrosative signaling), NOS/VEGF gene expression).

Therefore, the evidence showed that BPC 157 therapy can compensate, at least partly, for the function of the removed adrenal gland (i.e., BPC 157 therapy has a notable general effect on adrenalectomy-occlusion/occlusion-like syndrome). Likewise, it can rapidly recover the failed function of the other adrenal gland. Even with the molecular readouts as supportive rather than definitive mechanistic evidence, these occurred in a particular vascular way, likely involving many special pathways, and distinctive effects, and NO-system, in particular.

## 3. Discussion

This study of BPC 157 efficacy in unilateral adrenalectomy suggests a particular, rapidly occurring cytoprotective link, adrenalectomy → adrenal remnant dysfunction → generalized vascular occlusion-like syndrome. Given particular “vascular fragility” as an inherent cytoprotection failure in adrenalectomized rats [3], this novel evidence, unilateral adrenalectomy-induced occlusion/occlusion-like syndrome, may be a novel conceptual proof. In particular, this may be the very early post-operative intervals, which are so far much less investigated. Likewise, previously noted BPC 157 therapeutic effect in the FTIR vascular disturbance studies [15,16] appears likely to be extended.

Consequently, considering the applied extensive methodology approach, there is an indicative essential FTIR mechanistic background preceding this unilateral adrenalectomy study [15,16]. ECM degradation, lipid peroxidation, and GAG loss were reversed. Likewise, there is a general significance of the BPC 157 counteracting potential in the adrenalectomized rats’ aorta [16]. Such a direct vascular rescuing effect in FTIR studies occurs immediately [16]. Thus, there is an additional considerable impact of unilateral adrenalectomy: severe vascular and multiorgan failure, occlusion/occlusion-like syndrome and the stable gastric pentadecapeptide BPC 157 therapy. Conceptually (i.e., failed cytoprotection ≈ pleiotropic lesions (↑occlusion/occlusion-like syndrome) vs. cytoprotection → organoprotection (via therapy) (↓occlusion/occlusion-like syndrome), the course for this novel essential point had been well prepared [16].

Accordingly, the well-established basis [16] for this novel concept may reveal that unilateral adrenalectomy leads in this very early period (at 15 min, 5 h, 24 h) to the highly integrated downhill course. These are gross and morphological changes, blood pressure disturbances, thrombosis, hemorrhage, peripherally and centrally, vascular alterations, arrhythmias, oxidative stress parameters, molecular markers, and occlusion/occlusion-like syndrome. Notably, this very breadth may also become a weakness in the current report. The question could be about particularities of the assessment for each of these parameters, although used as approved in the previous studies [17,18,19,20,21,22,23,24,25,26,27,28,29,30]. This would be the pathology-framework. Thought to be the most suited for a multi-organ study [17,18,19,20,21,22,23,24,25,26,27,28,29,30], this would be the used unified semiquantitative assessment across all organs, added quantitative (i.e., adrenal gland, brain) and qualitative description. Specifically, for the mechanistic part carried out in accordance with our previous [71,72,73,74] and other studies [75,76,77,78,79], this would be PCR reactions performed in duplicate [71,72,73,74,75,76,77,78,79] using GAPDH as a housekeeping gene [80,81]. However, taken all together, with methodology previously approved [17,18,19,20,21,22,23,24,25,26,27,28,29,30], they appear in a sequence, both locally and systemically, as a tightly interconnected network of consistent interrelated supporting evidence. Thus, matching evidence after unilateral adrenalectomy, the severe vascular and multiorgan failure, as an occlusion/occlusion-like syndrome, is a consistent and reproducible experimental model. Consequently, in these early periods after unilateral adrenalectomy, the consistent evidence of counteraction supports BPC 157 as useful therapy, as BPC 157 therapy had resolved other occlusion/occlusion-like syndromes [17,18,19,20,21,22,23,24,25,26,27,28,29,30].

This implies, in terms of cytoprotection [31,32,33,34,35,36,37,38,39,40,41,42] and BPC 157 terms [17,18,19,20,21,22,23,24,25,26,27,28,29,30], that adrenal gland function or dysfunction underlies the occlusion/occlusion-like syndrome after major injuries [17,18,19,20,21,22,23,24,25,26,27,28,29,30], in general, and in its development and resolution with BPC 157 therapy [17,18,19,20,21,22,23,24,25,26,27,28,29,30], in particular. A general dysfunction, due to a general cytoprotection failure, means simultaneous lesions in different organs [31,32,33,34,35,36,37,38,39,40,41,42], severe vascular, and multiorgan failure, as evidenced in the present report. Promptly repelled by application of BPC 157 as a cytoprotection agent means an essential key to reestablish cytoprotection function (cytoprotection → organoprotection) [17,18,19,20,21,22,23,24,25,26,27,28,29,30]. As in our previous studies [4], vascular recovery (recently specifically pointed out in FTIR studies [15,16]) is with particular effects on the NO-system and oxidative stress. There are increased NO levels in all tissues, and increased NOS1, NOS2, and NOS3 expression and VEGF-A gene expression in the adrenal, along with strong counteraction of the increased MDA levels in all tissues investigated. Thus, the previously evidenced NO-system modulation/oxidative balance [4] accordingly occurs after BPC 157 therapy in adrenalectomized rats. This important combined effect of BPC 157 therapy (↑NO/↓MDA) is used as a safety key alongside BPC 157 therapy [4]. A special beneficial pleiotropic effect means controlling and modulating angiogenesis and the NO-system [4]. Otherwise, the unopposed increased MDA levels indicate a perilous chain of events [4]. This was associated with its function counteracting the adverse effects of NO-blockade (L-NAME-hypertension, and procoagulant effect), and adverse effects of NO-overactivity (L-arginine-hypotension and anticoagulant effect) [67,68,69,70]. Likewise, it maintains thrombocytes without affecting coagulation pathways (i.e., aggregometry and elastometry studies) [69,82,83]. All these might be its modulatory effects, interaction with several molecular pathways [56,57,58,59,60,61,62,63,64,65,66], i.e., NO-pathways, the VEGFR2-Akt-eNOS and Src-Caveolin-1-eNOS signaling pathways controlling vasomotor tone [58,59,60]. Accordingly, as emphasized before as a proof of direct cytoprotective vascular effect, in BPC 157 therapy, FTIR studies demonstrated a direct reversing effect on disturbed molecular signatures on the vessel wall in adrenalectomy-induced vascular injury [15,16]. This may connect its effect close to counteraction of the noxious chain of events leading to impaired vessel viscoelasticity and barrier function [15,16]. Likewise, this was associated with its function as a free radical scavenger and stabilizer of the cellular junction [56,57], leading to the significantly mitigated leaky gut syndrome [57]. There, as the most recent evidence, combining animal findings with presentation in human tissue, a study in the human internal mammary artery indicates that similar BPC 157/NO relations could occur in humans [52].

Therefore, the resolution of adrenal lesions and functions includes the whole chain of events and coincides with a plethora of interrelated courses, previously evidenced as well [4,5,6].

As emphasized [4,5,6], in adrenalectomized BPC 157 rats, there was collateral pathway activation, i.e., azygos vein direct blood flow delivery, reversal of the collapsed peduncle of the inferior suprarenal artery, and superior suprarenal vein. Likewise, there was counteraction of the lesions in the brain (intracerebral, intraventricular hemorrhage), heart (congestion, arrhythmias), lung (congestion, intra-alveolar hemorrhage), and severe congestion in the liver, kidney, and gastrointestinal tract. Consistently, there was the counteraction of the progressing thrombosis, peripherally and centrally, arrhythmias, vessel failure (congested inferior caval vein and superior mesenteric vein appeared close to normal), attenuated/eliminated blood pressure disturbances, intracranial (superior sagittal sinus), portal, caval hypertension, and aortic hypotension, and advanced Virchow triad circumstances reversal. Thus, the therapy effect in adrenalectomized rats consistently appeared as in other occlusion/occlusion-like syndrome recoveries (i.e., counteraction of Virchow triad circumstances commonly occurred) [17,18,19,20,21,22,23,24,25,26,27,28,29,30].

Novel points perceive the harmful condition of adrenalectomy-occlusion/occlusion-like syndrome that can be barely sustained. The removal of the adrenal gland to induce occlusion/occlusion-like syndromes interrelates all of these events, initiation, and reversal, to the adrenal gland dysfunction or to the adrenal gland recovery, respectively. Local (adrenal gland) and systemic (occlusion/occlusion-like syndrome) events concurrently occurred. As an immediate and persistent highlight, there is the reversal of the collapsed peduncle of the inferior suprarenal artery and superior suprarenal vein to the remaining adrenal gland recovery. It occurred rapidly. Notably, attenuated/eliminated were both lipid depletion (zona glomerulosa and fasciculate) and congestion (medulla). These suggest in sequence counteraction of the general adrenal gland insufficiency, reversal of extensive adrenal congestion, reversal of more limited adrenal storage, and reversal of more limited cortisol response (i.e., cortisol is synthesized from cholesterol). Diffuse lipid depletion indicates the loss of cholesteryl esters in the adrenal glands [84,85,86], and in the zona fasciculate as the common hallmark of severe circumstances in both humans and animals [87,88]. Considering that this occurs along with compensatory adrenal growth, this response has to be highly specific (not blocked by hypophysectomy or glucocorticoid administration).

This suggests that BPC 157 therapy can specifically act as effective therapy supporting organ function and vascular stability despite low cortisol rather than directly replacing cortisol. Likely, in this way, BPC 157 therapy can substitute the still-limited cortisol response and can be commenced to prevent death from adrenal insufficiency. However, immediately, with BPC 157 therapy, this can take over the function of the disabled major vessel, to compete with the progressing Virchow in the post-adrenalectomy syndrome as well, as to bring direct blood flow to the superior caval vein via activated azygos vein, to compensate for the vascular failure and reestablish reorganized blood flow [17,18,19,20,21,22,23,24,25,26,27,28,29,30].

At the general occlusion/occlusion-like syndrome and unilateral adrenalectomy occlusion/occlusion-like syndrome level, at the same time, the shared rapid recovery of the major vessels may have particular importance. There, the vascular rescue (i.e., the congested inferior caval vein and superior mesenteric vein recovered to normal vessel presentation, the collapsed abdominal aorta recovered, and the activated azygos vein direct blood flow delivery occurred as the main rescuing pathway) may suggest a context-dependent beneficial effect. Thereby, in adrenalectomized rats, the heart dysfunction (i.e., congestion and dilatation of coronary arteries and arrhythmias (tachycardias and shortened QT interval)) accords with the substantiated recovery of disabling heart failure as a whole [17,18,19,20,21,22,23,24,25,26,27,28,29,30]. This can be since therapy’s effect on myocardial infarction, heart failure, pulmonary hypertension, arrhythmias, and thrombosis presentation was reviewed [4,5,6]. Note, these arrhythmias were counteracted as they were counteracted in amphetamine rats, otherwise associated with increased levels of catecholamine (amphetamine) that can degenerate into paroxysmal atrial fibrillation and/or ventricular tachycardia, potentially leading to sudden cardiac death [30]. Thus, there is an indicative demonstration of BPC 157 particular antiarrhythmic effect, which was recently reviewed as a well-matched cytoprotective-antiarrhythmic effect [6]. This is again the evidence that BPC 157 has a conditional antiarrhythmic effect, successfully applicable in those particular conditions, and not a constitutive effect as standard antiarrhythmics [6].

Likely, this means that also in adrenalectomized rats, BPC 157 therapy may ascertain the ability to drain venous blood adequately (i.e., in occlusion/occlusion-like syndromes [17,18,19,20,21,22,23,24,25,26,27,28,29,30] and in adrenalectomized rats as well, the intra-alveolar hemorrhage did not occur in BPC 157 therapy-treated rats [17,18,19,20,21,22,23,24,25,26,27,28,29,30]). Thus, for BPC 157 therapy in adrenalectomized rats, there is a reversal of a harmful inability to drain venous blood adequately for a given cerebral blood inflow without raising venous pressures. There is an attenuation/elimination of intracranial (superior sagittal sinus) hypertension, portal and caval hypertension, and aortal hypotension. These effects in BPC 157 therapy in adrenalectomized rats simultaneously appeared and verified each other’s effect as the brain swelling grossly was rapidly attenuated [17,18,19,20,21,22,23,24,25,26,27,28,29,30]. Therefore, it can be suggested that brain presentation (immediately and persistently swollen brain, its aggravation, vs. reversal based on the BPC 157 therapy’s beneficial effects) can illustrate high therapy potential. This could be against a perilous range of lesions and disturbances, a poor balance, inability to sustain any further challenge, and full functional inability. Amid intraperitoneal or intragastric saline administration, the additional brain swellings are a perilous inability sign, also shown, illustrating full disability in rats with occluded superior sagittal sinus [20].

Thus, we evidence that in these early periods after unilateral adrenalectomy, even with low cortisol level, BPC 157 therapy can compensate, at least partly, for the function of the removed adrenal gland (i.e., BPC 157 therapy has a notable general effect on adrenalectomy-occlusion/occlusion-like syndrome), and can rapidly recover the failed function of the other adrenal gland. This can also be perceived in general stress terms and suggests BPC 157’s role in the stress response [89]. The adrenal gland is the key effector organ involved in the activation of the hypothalamic-pituitary-adrenal (HPA) axis [90]. After adrenalectomy, the remaining adrenal gland is the essential breaking point, whether regularly malfunctioning or recovering function through therapy (note, BPC 157 therapy might provide a particular central/peripheral equation [91], and thereby, counteract both the adrenal and peripheral organ lesions and brain lesions and hemorrhage, in particular, that in the hypothalamus). Additionally, BPC 157 therapy counteracted systemic corticosteroid-impaired healing in many tissues (i.e., skin, tendon, muscle, and gastrointestinal tract), and inhibited corticosteroid immunosuppression in vitro [4]. In general, if an agent inhibits corticosteroid effects, this strongly suggests that the agent is interacting—directly or indirectly—with the adrenal corticosteroid system or with downstream glucocorticoid signaling pathways [92,93,94,95,96,97,98]. BPC 157 administration counteracted the disabling effect on adrenals of the aniline [99], a steroid synthesis inhibitor [84,85,100], and aniline-adrenal lesions comparable to congenital lipoid adrenal hyperplasia in newborn infants [101,102,103,104,105,106].

Finally, within the unilateral adrenalectomy-occlusion/occlusion-like syndrome, we could envisage specific targets resolved by BPC 157 therapy and newly revealed BPC 157/adrenal gland relations. Notably, these BPC 157 therapies and BPC 157/adrenal gland relations remain to be seen within the scope of reviews of other groups focused on BPC 157 healing efficacy [90,107,108,109,110,111,112,113]. Moreover, in recent years, in particular soft tissue injuries and BPC 157 efficacy have appeared in the focus of a growing number of reviews [114,115,116,117,118,119,120,121,122,123,124,125,126,127,128,129,130,131,132,133,134,135,136]. Furthermore, the evidence derived from the majority of experimental work (i.e., initial discovery, mechanistic exploration, and model validation) [4,5,6,91,99] originating from a single research group stands with the confirmatory reports of other groups (i.e., [47,48,56,57,58,59,60,61,62,63,64,137,138,139,140,141,142,143,144,145,146,147,148,149]). Translational values, in general, stand with the similar therapeutic effect across different routes of administration in the same model in many studies [4,5,6,91,99]. In addition, BPC 157 induces concentration-dependent vasorelaxation in human arterial tissue, predominantly mediated via an endothelium-dependent NO pathway [52]. Translation value of the cytoprotection concept, as originally defined [31,32,33,34,35,36,37,38,39,40,41,42], means extending it from local epithelial/endothelial protection to systemic endothelial stabilization and multiorgan protection.

Thus, in the adrenalectomy issue, this provides a substantial, although still developing, novel framework. This framework may substantiate BPC 157 therapy and BPC 157/adrenal gland relations in adrenalectomized rats. This encompasses the integrated gross and morphological changes, blood pressure disturbances, thrombosis, hemorrhage, peripherally and centrally, vascular alterations, arrhythmias, oxidative stress parameters, molecular markers, and occlusion/occlusion-like syndrome, analyzed separately or together. This could be even with the molecular readouts are presented explicitly as supportive rather than definitive mechanistic evidence. With the adrenal gland, this might be seen as a network of cytoprotective evidence (i.e., pleiotropic beneficial effects). This could be for the physiologic significance of the revealed BPC 157/NO-system interplay [4] (i.e., BPC 157 was found in in situ hybridization and immunostaining studies in humans to be largely distributed in tissues [150]). It may have additional physiologic regulatory roles, i.e., released from the stomach and sent to other organs [4,5,6,72,91,99,150]. These points, and further application, are supported by its efficacy in clinical trials (although limited) and no adverse effects in clinical trials and in toxicology studies [4,43,44,45,46,47,48,49,50,51]. Notably, following demonstration of its direct reversing effect on disturbed molecular signatures on the vessel wall in FTIR studies [15,16], this report again focused on the most important early period following adrenalectomy. Within this framework, we propose that unilateral adrenalectomy induces a syndrome with features resembling an occlusion/occlusion-like condition, a model-based interpretation within a defined time frame. Therefore, given its potential applicability in adrenal and related disorders, further studies are warranted. However, in support, regardless of possible limitations of this study, BPC 157 was effective given in the range of 10 µg–10 ng/kg, including the peroral route, as before [17,18,19,20,21,22,23,24,25,26,27,28,29,30]. It exhibits a harmless limit test, 2 g/kg i.v. or i.g., without adverse effects in mice. LD1 was not achieved [4,5,6,72,91,99,150], evidence fully confirmed in other studies [47,48].

## 4. Materials and Methods

### 4.1. Animals

The study was conducted with appropriately randomized male albino Wistar rats, 12–16 weeks of age, 280 g in body weight, who were self-breeded in the Department of Pharmacology, Faculty of Medicine, Zagreb, Croatia. The facility for animals was registered by the Veterinary Directorate (Reg. No.: HR-POK-007). Laboratory rats were acclimatized for five days and assigned identification numbers prior to allocation. Randomization was performed using a computer-generated random number sequence with block randomization to ensure equal group sizes (*n* = 6 animals per group per time point) across all treatments and postoperative intervals. The laboratory animals were housed in polycarbonate (PC) cages in conventional laboratory conditions at 20–24 °C, relative humidity of 40–70%, and a noise level of 60 dB. The cages were identified with the dates, study number, group, dose, number, and sex of each animal. Twelve-hour daylight was provided by fluorescent lighting. They received standard nutrition (pelleted feed) and fresh water by free access (ad libitum) in accordance with Good Laboratory Practice (GLP). The care of the animals was in accordance with the standard operating procedures of the facility for pharmacological animals and the European Convention for the Protection of Vertebrate Animals Used for Experimental and Other Scientific Purposes (ETS 123). This research was approved by the local Ethics Committee (case number 380-59-10106-17-100/290; Approval Date: 30 October 2017) and by the Directorate of Veterinary (UP/I-322-01/15-01/22). The ethical principles of the study were in accordance with the European Directive 2010/63/EU, the Act on Amendments to the Animal Protection Act (Official Gazette 37/13), the Animal Protection Act (Official Gazette 135/06), the Ordinance on the Protection of Animals Used for Scientific Purposes (Official Gazette 55/13), the recommendations of the Federation of European Laboratory Associations for Animal Science (FELASA), and the recommendations of the Ethics Committee of the Faculty of Medicine, University of Zagreb. The experiments were evaluated by an independent observer who was blinded to the treatment allocation.

An a priori power analysis was conducted for a representative primary outcome (liver lesion score) using a two-sided significance level (α = 0.05) and a statistical power of 80% (1 − β = 0.80). Based on effect sizes observed in previous studies using the same experimental model, a large standardized effect size (Cohen’s d = 1.2) was assumed. Under these assumptions, the minimum required sample size was calculated as n = 6 animals per group per time point to detect statistically significant differences between groups. Accordingly, this group size was applied consistently across all experimental conditions and time points. The study was designed as a multifactorial experimental model with repeated independent cohorts across time points (15 min, 5 h, 24 h), rather than a single repeated-measures design. Therefore, each time point was treated as an independent experimental comparison, with identical group allocation and sample size, ensuring statistical independence across cohorts.

### 4.2. Drugs

Stable gastric pentadecapeptide BPC 157 (GEPPPGKPADDAGLV, molecular weight 1419; Diagen, Ljubljana, Slovenia), a partial sequence of the human gastric juice protein BPC, which is freely soluble in water at pH 7.0 and in saline, was prepared as a peptide with 99% high-performance liquid chromatography (HPLC) purity, with 1-des-Gly peptide being the main impurity. The BPC 157 dose and application regimens (10 µg or 10 ng/kg given as an intragastric administration), were as described previously (i.e., without the use of a carrier or peptidase inhibitor) (for review see, i.e., [4,5,6,91,99,150]).

### 4.3. Experimental Protocol

Surgical removal of the left adrenal gland was performed under total anesthesia (intraperitoneal (ip), 40 mg/kg thiopental (Rotexmedica, Trittau, Germany) and 10 mg/kg diazepam (Apaurin; Krka, Novo Mesto, Slovenia)). Unilateral adrenalectomy was accomplished via the classic dorsal approach [1]. Sacrifice was at 15 min (i), 5 h (ii), and 24 h (iii). Rats received therapy BPC 157 (10 µg or 10 ng/kg) or saline (5 mL/kg) (controls) as an early intragastric regimen at 5 min upon adrenalectomy. Complete calvariectomy was carried out either at 15 min before adrenalectomy (sacrifice at 15 min post-adrenalectomy time (i)) or at 15 min before sacrifice (sacrifice at 5 h (ii) or 24 h (iii) post-adrenalectomy time) using the method described before [17,18,19,20,21,22,23,24,25,26,27,28,29,30]. The procedure included medially to the superior temporal lines and temporalis muscle attachments, 6 burr holes drilled in three horizontal lines (just basal from the posterior interocular line (two rostral burr holes); just rostral to the lambdoid suture (and transverse sinuses) on both sides (two basal burr holes); in line between the basal and rostral burr holes (two middle burr holes)). Recordings was with a camera attached to a VMS-004 Discovery Deluxe USB microscope (Veho, Claymont, DE, USA) brain swelling, azygos vein, superior mesenteric vein, portal vein, inferior caval vein, abdominal aorta, peduncle of the inferior suprarenal artery and superior suprarenal vein, heart and corresponding organ lesions). The time-line procedure previously used in our vascular studies was carried out (i.e., before the procedure, after adrenalectomy, after therapy application, and before sacrifice depending about the each of used protocols (i, ii, iii)) [17,18,19,20,21,22,23,24,25,26,27,28,29,30].

### 4.4. Superior Sagittal Sinus, Portal, and Caval Vein, and Abdominal Aorta Pressure Recording

Recordings followed the procedure used and described in details in our previous vascular studies [17,18,19,20,21,22,23,24,25,26,27,28,29,30], (deeply anesthetized rats, a cannula (BD Neoflon™ Cannula) connected to a pressure transducer (78534C MONITOR/TERMINAL; Hewlett Packard, Palo Alto, CA, USA), inserted into the portal vein, inferior caval vein and superior sagittal sinus, as well as the abdominal aorta at the level of the bifurcation at 15 min (i), 5 h (ii), and 24 h after adrenalectomy. The superior sagittal sinus anterior part was cannulated using a Braun intravenous cannula’ then, after laparotomy, the pressure recording in the portal vein, inferior vena cava, and abdominal aorta was performed.

Accordingly [17,18,19,20,21,22,23,24,25,26,27,28,29,30], superior sagittal sinus pressure of −24 to −27 mmHg, portal pressure of 3–5 mmHg similar to that of the inferior vena cava, though with values at least 1 mmHg higher in the portal vein, and abdominal aorta blood pressure values 100–120 mm Hg at the level of the bifurcation, were considered as normal in healthy rats. Notably, as shown before [17,18,19,20,21,22,23,24,25,26,27,28,29,30], these procedures produced no lesion in rats (sham animals).

### 4.5. ECG Recording

ECGs were recorded continuously in deeply anesthetized rats for all three main leads, by positioning stainless steel electrodes on all four limbs using an ECG monitor with a 2090 programmer (Medtronic, Minneapolis, MN, USA) connected to a Waverunner LT342 digital oscilloscope (LeCroy, Chestnut Ridge, NY, USA) (before procedure, at 15 min, 5 h and 24 h after adrenalectomy). This arrangement enabled precise recordings, measurements, and analysis of ECG parameters [17,18,19,20,21,22,23,24,25,26,27,28,29,30].

### 4.6. Thrombus Assessment

Following sacrifice, the superior sagittal sinus, and peripherally the portal vein, inferior caval vein, and abdominal aorta were removed from the rats, and the clots were weighed [17,18,19,20,21,22,23,24,25,26,27,28,29,30].

### 4.7. Cortisol Assessment

The Roche Elecsys cortisol assay was a competitive electrochemiluminescence immunoassay performed on Roche cobas analyzer e801 (Roche Diagnostics GmbH, Mannheim, Germany). The assay has a claimed measuring range of 1.5–1750 nmol/L, a limit of quantitation of 3.0 nmol/L, an inter-assay precision (CV) of 1.9% (at 310 nmol/L) and 2.1% (at 734 nmol/L). In rats with intact both adrenals, normal values were as follows 221 ± 28 (before surgery), and after a sham procedure, at 15 min, 331 ± 28, at 5 h, and at 24 h 304 ± 33 values comparable to the reported data [151].

### 4.8. Volume Presentation of the Brain, Heart, and Vessel

We applied the procedure used before in our previous vascular studies [17,18,19,20,21,22,23,24,25,26,27,28,29,30]. Brain volume and vessel volume and heart volume were proportional to the change in the brain or vessel or heart surface area. The presentation of the brain and peripheral vessels (superior mesenteric vein, inferior caval vein, azygos vein, abdominal aorta, and peduncle of the inferior suprarenal artery and superior suprarenal vein) and heart was recorded in deeply anesthetized rats, with a camera attached to a VMS-004 Discovery Deluxe USB microscope (Veho, Claymont, DE, USA) [17,18,19,20,21,22,23,24,25,26,27,28,29,30]. The border of the brain (or vessels, or heart) in the image was marked using ImageJ software and then the surface area of the brain (or veins, or heart) was measured. This was done with brain (or veins or heart) images for healthy rats, and then for both the control (saline) group and treated (BPC 157) group of rats at the same intervals after the application and at the time of sacrifice. The arithmetic mean of the surface areas was calculated for both groups. Then, the ratio of these two areas was calculated as ($\frac{A_{con}}{A_{bpc}}$), where Acon is the arithmetic mean brain (or veins or heart) area of the control group and Abpc is the arithmetic mean brain (or veins or heart) area of the treated group. Starting from the square-cube law Equations (1) and (2), an equation for the change in brain (or vessels, or heart) volume proportional to the change in brain (or vessels, or heart) surface area (6) was derived. In expressions (1)–(5), *l* is defined as any arbitrary one-dimensional length of the brain (for example rostro-caudal length of the brain), used only for defining the one-dimensional proportion (l2/l1) between two observed brains (or veins or heart) and as an inter-factor (and because of that not measured (6)) for deriving final expression (6). The procedure was as follows:

(1) $$A_{2}=A_{1}\times {\left(\frac{l_{2}}{l_{1}}\right)}^{2}\;(\mathrm{square}\text{-}\mathrm{cube}\;\mathrm{law})$$

(2) $$V_{2}=V_{1}\times {\left(\frac{l_{2}}{l_{1}}\right)}^{3}\;(\mathrm{square}\text{-}\mathrm{cube}\;\mathrm{law})$$

(3) $$\frac{A_{2}}{A_{1}}={\left(\frac{l_{2}}{l_{1}}\right)}^{2}$$

from (1), after dividing both sides by A1,

(4) $$\frac{l_{2}}{l_{1}}=\sqrt{\frac{A_{2}}{A_{1}}}$$

from (3), after taking the square root of both sides,

(5) $$\frac{V_{2}}{V_{1}}={\left(\frac{l_{2}}{l_{1}}\right)}^{3}$$

from (2), after dividing both sides by V1

(6) $$\frac{V_{2}}{V_{1}}={\left(\sqrt{\frac{A_{2}}{A_{1}}}\;\right)}^{3}$$

after incorporating expression (4) into Equation (5).

### 4.9. Gross Assessment of Gastrointestinal Lesions

For recording, we used a camera attached to a VMS-004 Discovery Deluxe USB microscope (Veho, Claymont, DE, USA). As described before, gross lesions in the gastrointestinal tract and in the stomach (sum of the longest diameters, mm) were assessed in deeply anaesthetized rats, laparatomized before sacrifice [17,18,19,20,21,22,23,24,25,26,27,28,29,30].

### 4.10. Microscopy

As described in the previous studies [17,18,19,20,21,22,23,24,25,26,27,28,29,30], evaluation was by light microscopy using an Olympus 71 digital camera and an Olympus BX51 microscope (OLYMPUS Europa SE&CO.KG, Hamburg, Germany). Digital images were saved as uncompressed 24-bit RGB TIFF files using the software program AnalySIS (Olympus Soft Imaging System GmbH, Munster, Germany). Representative tissue specimens (i.e., the brain, heart, lungs, liver, kidney, stomach, small intestine and adrenal gland taken at the end of the experiment, fixed in 10% neutral buffered formalin (pH 7.4) at room temperature for 24 h) were embedded in paraffin, sectioned at 4 μm, stained with hemalaun and eosin (H&E). Thought to be the most suited for a multi-organ study [17,18,19,20,21,22,23,24,25,26,27,28,29,30], pathological assessment was based on the predefined semiquantitative assessment across all organs, added quantitative (i.e., adrenal gland, brain) and qualitative description.

#### 4.10.1. Brain Histology

As described in the previous studies [17,18,19,20,21,22,23,24,25,26,27,28,29,30], the brain was dissected according to NTP-7, at Level 3 and 6 with neuroanatomic subsites presented in certain brain sections using coronal sections with three mandatory sections. We used a semiquantitative neuropathological scoring system, and the sum of analyzed affected areas (0–4) (i) and karyopyknotic cells in the brain areas (0–4) (ii) making (i) + (ii) a combined score (0–8), as follows. (i). Specifically affected brain areas (cerebral (NTP-7, Level 3), cerebellar cortex (NTP-7, Level 6), hippocampus, thalamus, and hypothalamus (NTP-7, Level 3)) were scored (0–4), (score 0 indicates no histopathologic change), as follows. Small, patchy, complete or incomplete infarcts (≤10% of the area affected) represented score 1. Partly confluent or incomplete infarcts (20–30% of the area affected) represented score 2. Large confluent complete infarcts (40–60% of the area affected) represented score 3. In the cortex total disintegration of the tissue, in the hypothalamus, thalamus, and hippocampus large complete infarcts (>75% of the area affected) represented score 4. (ii). Analyzed were karyopyknotic cells in the affected brain areas (0–4) (score 0 indicates no change), cerebral (NTP-7, Level 3), cerebellar cortex (NTP-7, Level 6), hippocampus, thalamus, and hypothalamus (NTP-7, Level 3) as follows: a few karyopyknotic of neuronal cells (≤20%) (score 1); patchy areas of karyopyknotic cells (50%) (score 2); more extensive karyopyknotic areas (75%) (score 3); complete infarction (100%) (score 4). Brain tissue hemorrhage was obtained by estimating the percentage of affected areas. Intraventricular hemorrhage was noted as present or absent.

We also assessed the neuronal pathological changes in acquired digital images saved as uncompressed 24-bit RGB TIFF files in the software program AnalySIS (Olympus Soft Imaging System GmbH, Munster, Germany) performing quantitative analysis of neuronal damage in the karyopyknotic areas. The neurons of the cortical cerebral, cerebellar region, hippocampus, and hypothalamus were counted in 10 different high-powered fields (HPF, 400×), and 3 to 5 serial sections of each sample were used to do the count as described [152]. The field size was 0.24 μm^2^.

We used four criteria for the estimation of the edema: pale myelin, sieve-like appearance of myelinated areas, dilation of perivascular and pericellular spaces, and vacuolar appearance of the neuropil of gray matter. Edema was graded as heavy, moderate, slight, or no edema (score 0–3) [153].

#### 4.10.2. Lung Histology

The same scoring system as in the previous studies [17,18,19,20,21,22,23,24,25,26,27,28,29,30] was used to grade the degree of lung injury in lung tissue analysis. Each of the features (i.e., focal thickening of the alveolar membranes, congestion, pulmonary edema, intra-alveolar hemorrhage, interstitial neutrophil infiltration, and intra-alveolar neutrophil infiltration) was scored (0–3) as absent (0) or present a mild (1), moderate (2), or severe (3) degree and a final histology score was determined.

#### 4.10.3. Renal, Liver, and Heart Histology

The same scoring system as in the previous studies [17,18,19,20,21,22,23,24,25,26,27,28,29,30] was used to grade renal (i.e., the degeneration of Bowman’s space and glomeruli, degeneration of the proximal and distal tubules, vascular congestion, and interstitial edema), liver (i.e., vacuolization of hepatocytes and pyknotic hepatocyte nuclei, activation of Kupffer cells, and enlargement of sinusoids) and heart (i.e., dilatation and congestion of blood vessels within the myocardium and coronary arteries) histology. Each specimen was scored using a scale ranging from 0–3 (0: none, 1: mild, 2: moderate, and 3: severe) for each criterion, and a final histology score was determined (0: none, 1: mild, 2: moderate, and 3: severe).

#### 4.10.4. Gastrointestinal Histology

As in previous studies [17,18,19,20,21,22,23,24,25,26,27,28,29,30], we used a histologic scoring scale adapted from Chui and coworkers [154] for the stomach tissue damage scoring 0–5 (normal to severe) in three categories (mucosal injury, inflammation, hyperemia/hemorrhage) for a total score of 0 to 15, as described by Lane and coworkers [155]. Illustratively, the assessment included morphologic features of mucosal injury (i.e., different grades of epithelial lifting, villi denudation, and necrosis), inflammation (i.e., focal to diffuse according to lamina propria infiltration or subendothelial infiltration), and hyperemia/hemorrhage (i.e., focal to diffuse according to lamina propria or subendothelial localization).

#### 4.10.5. Adrenal Gland

Left adrenals were weighed (adrenal gland weight per 100 g of total body weight) before they were put in 10% formaldehide solution. After sacrificing animals, the right adrenals were collected, freed from the adjacent adipose tissue, weighed (adrenal gland weight per 100 g of total body weight), cut in half and stored in vials at −70 °C for mRNA, oxygen radical and NO analysis and in 10% formaldehide solution for pathohistological assessment. In the adrenal gland zona glomerulosa, fasciculata, and reticularis % loss of lipid vacuoles was calculated. In the adrenal gland medulla, congestion score was determined (0: none, 1: mild, 2: moderate, and 3: severe).

### 4.11. Oxidative Stress in the Samples

The assessment was at 15 min, 5 h, and 24 h. In the tissue samples, adrenal, stomach and brain, we assessed oxidative stress by quantifying the thiobarbituric acid (TBA) reactivity as malondialdehyde equivalents (MDA). Collected tissues were homogenized in ice cold phosphate buffer (pH 7.5) using glass pestle. We centrifuged the samples (3000× *g* rpm, 5 min), and collected the supernatants. Trichloroacetic acid (TCA 10%, incubation on ice for 30 min) was added to precipitate proteins, remove contaminants, and inhibit protease activity. (Samples were centrifuged (12,000× *g* rpm, 5 min) and supernatants stored at −80 °C until further processing (MDA and NO determination). Then, we added 1% TBA and boiled the samples (95 °C, 60 min). We maintained the tubes in ice for 10 min and determined the absorbance at the wavelengths of 532 and 570 nm. We determined MDA concentration from the standard calibration curve plotted using 1,1,3,3′-tetra-ethoxy propane (TEP). We expressed the lipid peroxidation extent as the concentration of MDA, using a molar extinction coefficient for MDA of 1.56 × 105 mol/L/cm. The results are expressed in nmol/mg of protein.

### 4.12. NO Determination in the Samples

The assessment was at 15 min, 5 h and 24 h. Griess reaction (Griess Reagent System, Promega, Madison, WI, USA) was used for the determination of the NO levels in the adrenal, stomach and brain samples. To supernatants prepared as described above, we added sulfanilamide per manufacturer instructions. Tissue was incubated, and then, we added N-(1-naphthyl)ethylenediamine dihydrochloride. The Griess reaction takes the diazotization reaction (i.e., acidified nitrite reacts with diazonium ions; then, it is coupled to N-(1-naphthyl)ethylenediamine dihydrochloride to form a chromophoric azo derivative). Sodium nitrite solution was the standard to measure the absorbance at 540 nm. The NO levels are expressed in µmol/mg protein. A commercial kit (BioRad Protein DR Assay Reagent Kit, Sigma-Aldrich, St. Louis, MO, USA) was used for the determination of the protein.

### 4.13. Gene Expression Analysis

Gene expression analysis was the acknowledged procedure [75,76,77,78,79], analyzed for BPC 157 in a wound healing review [72], and subsequently applied as before [71,73,74]. Relative gene expression was normalized to a single reference gene (GAPDH), consistent with published RT-qPCR analyses that have successfully utilized a single, experimentally validated internal control for normalization when stability across conditions is confirmed [80,81]. The tissue (adrenal gland) was rapidly dissected and frozen in liquid nitrogen. This was done after sacrifice at 15 min, 5 h, and 24 h after unilateral adrenalectomy and application of the saline (5 mL/kg i.g.) or BPC 157 (10 ng/kg i.g.). The tissue was disrupted using tissue homogenizer Bio-Gen PRO200 homogenizer (PRO Scientific, Oxford, CT, USA), in 1000 µL of TRIzol (Invitrogen, Thermo Fisher Scientific, Waltham, MA, USA) and the isolation itself was done using a TRIzol-based reagent method according to the manufacturer’s instructions. After RNA isolation step, nucleic acid concentration was measured with Nano Drop ND-1000 spectrophotometer (Nano DropTechnologies, Thermo Fisher Scientific, Waltham, MA, USA). Reverse transcription was performed using the High Capacity cDNA Reverse Transcription Kit (Applied Biosystems, Thermo Fisher Scientific, Waltham, MA, USA) following manufacturer’s instructions and using a ProFlex PCR System machine (Applied Biosystems, Thermo Fisher Scientific, Waltham, MA, USA). TaqMan Gene Expression Assay (Applied Biosystems, Termo Fisher Scientific, Waltham, MA, USA) hydrolysis probes were used for gene expression analysis of selected genes (Table 3) with TaqMan Gene Expression Master Mix (Applied Biosystems, Termo Fisher Scientific, Waltham, MA, USA). Quantitative PCR was carried out in duplicate for every sample. Reactions were performed with Cobas z 480 instrument (Hoffmann-La Roche Ltd., Basel, Switzerland) according to the following protocol: 2 min at 50 °C, 10 min at 95 °C, 45 cycles of 15 s at 95 °C and 1 min at 60 °C. We analyzed Gapdh as reference gene to normalize the results of several genes of interest: Vegfa, Nos1, Nos2, and Nos3. To determine the difference in gene expression between treated and non-treated samples, the formula 2-DDCt was used, where the DDCt is the difference between DCt of the treated sample and the DCt of the non-treated sample (Table 4).

### 4.14. Statistical Analysis

All quantitative data are expressed as mean ± standard deviation (SD). Raw individual measurements were available for all experimental endpoints and were used for all statistical analyses without exception. Each time point (15 min, 5 h, 24 h) was analyzed as an independent experimental cohort, consistent with the study design and a priori power analysis (*n* = 6 animals per group per time point). Data normality was assessed using the Shapiro–Wilk test, and homogeneity of variance using the Levene test. For datasets involving more than two experimental groups (control, BPC 157 10 µg/kg, and BPC 157 10 ng/kg), one-way ANOVA was applied, followed by Tukey’s post hoc test to control for multiple comparisons and Type I error. For non-normally distributed data, the Kruskal–Wallis test followed by Dunn’s multiple-comparison correction was used. For pairwise comparisons, the unpaired Student’s *t*-test (two-tailed) was applied for normally distributed data, while the Mann–Whitney U test was used when appropriate. Effect sizes were calculated to assess practical significance, using η^2^ (eta squared) for ANOVA and Cohen’s d for pairwise comparisons. Where applicable, 95% confidence intervals (CIs) for mean differences were determined.

Histological lesion scores and neuropathological indices were derived from predefined validated scoring systems and analyzed based on their full underlying distributions of individual values. Despite their ordinal origin, these variables were analyzed using parametric or non-parametric tests depending on the outcome of normality testing, in accordance with standard practice in experimental pathology, particularly in studies involving semi-quantitative lesion grading systems with biologically robust group separation. Given the large number of outcome measures across multiple organ systems, brain regions, and time points, detailed statistical outputs (including exact *p*-values, confidence intervals, and effect size ranges) are summarized in Table 3. This approach enables comprehensive and transparent reporting of statistical inference while maintaining clarity and readability of the main manuscript.

The availability of complete raw datasets enabled precise calculation of group means, variability, exact *p*-values, confidence intervals, and effect sizes. Across outcomes, consistency of findings was confirmed by convergent statistical results, with many parameters demonstrating strong group separation and correspondingly large effect sizes. In cases of near-complete or complete separation (minimal or zero variance), effect size estimates were interpreted with caution due to known instability of standardized metrics under such conditions.

All analyses were performed using GraphPad Prism version 10 (GraphPad Software, San Diego, CA, USA), and statistical significance was defined as *p* < 0.05.

## 5. Conclusions

A recent demonstration in a Fourier transform infrared spectroscopy vascular disturbance study provides a novel revealing insight. After unilateral adrenalectomy, in a very early post-adrenalectomy period (at 15 min, 5 h, 24 h), stable pentadecapeptide BPC 157 counteracted in the rat aorta severely disturbed molecular signatures of adrenalectomy-induced vascular injury (i.e., ECM degradation, lipid peroxidation, and loss of GAG content, leading to impaired vessel viscoelasticity and barrier function). Consequently, as an additional novel point, we demonstrated that unilateral adrenalectomy simultaneously induces a peripheral and central occlusion/occlusion-like syndrome in rats. Likewise, BPC 157, via its particular vascular recovery effect, counteracts unilateral adrenalectomy-induced occlusion/occlusion-like syndrome, recovers adrenal gland lesions, and restores function. This counteraction includes activation of the NO-system along with counteraction of the increased MDA levels, and direct effect on the vessel wall. This is simultaneously activating the azygos vein direct blood delivery to counteract multi-organ damage, and the recovered peduncle of the inferior suprarenal artery and superior suprarenal vein to rescue the adrenal gland. This expands the cytoprotection concept to include adrenal involvement in vascular failure states, BPC 157-unilateral adrenalectomy therapy to the counteraction of lesions in the adrenal, brain, heart, lung, liver, kidney, and gastrointestinal tract, arrhythmias, and attenuated/eliminated intracranial, caval, and portal hypertension and aortal hypotension, and thrombosis, peripherally and centrally. However, with all these findings, methodology and mechanistic considerations, as described before, further studies are warranted for BPC 157 therapy’s practical translation. On the other hand, there is also a very safe BPC 157 profile (i.e., no adverse effects in clinical trials (ulcerative colitis, phase II), and toxicological studies could not achieve a lethal dose (LD1) (for review see [4,5,6,72,91,99,150]).

## Figures and Tables

**Table 1:** Lesions, scored microscopically (heart, lung, kidney, stomach, small intestine, adrenal gland), or macroscopically (stomach), in rats at 15 min, 5 h, and 24 h following unilateral adrenalectomy. * *p* < 0.05, *at least*, vs. *control*.

Medication	Lesions Scored Microscopically (Heart, Lung, Kidney, Stomach, Small Intestine, Adrenal Gland), or Macroscopically (Stomach) in Rats at 15 min, 5 h, and 24 h Following Unilateral Adrenalectomy		
	15 min	5 h	24 h
	Heart (scored 0–3, Min/Med/Max)		
Saline	2/2/2	2/2/2	3/3/3
BPC 157 10 µg/kg i.g.	0/0/0 *	0/1/1 *	1/1/1 *
BPC 157 10 ng/kg i.g.	0/0/0 *	0/0/0 *	1/1/1 *
	Lung (scored 0–3, Min/Med/Max)		
Saline	2/2/2	2/2/2	3/3/3
BPC 157 10 µg/kg i.g.	1/1/1 *	1/1/1 *	1/1/1 *
BPC 157 10 ng/kg i.g.	1/1/1 *	1/1/1 *	1/1/1 *
	Liver (scored 0–3, Min/Med/Max)		
Saline	3/3/3	3/3/3	3/3/3
BPC 157 10 µg/kg i.g.	1/1/1 *	1/1/1 *	1/1/1 *
BPC 157 10 ng/kg i.g.	1/1/1 *	1/1/1 *	1/1/1 *
	Kidney (scored 0–3, Min/Med/Max)		
Saline	3/3/3	3/3/3	3/3/3
BPC 157 10 µg/kg i.g.	0/0/0 *	0/0/0 *	1/1/1 *
BPC 157 10 ng/kg i.g.	0/0/0 *	0/0/0 *	1/1/1 *
	Stomach (scored 0–15, Min/Med/Max)		
Saline	5/5/5	5/5/5	5/5/5
BPC 157 10 µg/kg i.g.	0/0/0 *	0/0/0 *	0/0/0 *
BPC 157 10 ng/kg i.g.	0/0/0 *	0/0/0 *	0/0/0 *
	Stomach lesion (sum of longest diameters, mean ± SD, mm)		
Saline	2.0 ± 0.2	3.0 ± 0.2	3.0 ± 0.2
BPC 157 10 µg/kg i.g.	0.0 ± 0.0 *	0.0 ± 0.0 *	0.0 ± 0.0 *
BPC 157 10 ng/kg i.g.	0.0 ± 0.0 *	0.0 ± 0.0 *	0.0 ± 0.0 *
	Small intestine (scored 0–15, Min/Med/Max)		
Saline	5/5/5	5/5/5	5/5/5
BPC 157 10 µg/kg i.g.	0/0/0 *	0/0/0 *	1/1/1 *
BPC 157 10 ng/kg i.g.	0/0/0 *	0/0/0 *	1/1/1 *
	Adrenal gland zona glomerulosa (mean ± SD, % loss of lipid vacuoles)		
Saline	80 ± 5	80 ± 5	20 ± 2
BPC 157 10 µg/kg i.g.	10 ± 1 *	3.5 ± 0.5 *	1.5 ± 0.5 *
BPC 157 10 ng/kg i.g.	10 ± 1 *	3.0 ± 0.5 *	1.0 ± 0.5 *
	Adrenal gland zona fasciculate (mean ± SD, % loss of lipid vacuoles)		
Saline	60 ± 5	55 ± 5	80 ± 5
BPC 157 10 µg/kg i.g.	60 ± 5	1.0 ± 0.5 *	1.0 ± 0.5 *
BPC 157 10 ng/kg i.g.	60 ± 5	1.5 ± 0.5 *	1.5 ± 0.5 *
	Adrenal gland medulla (scored 0–3, Min/Med/Max)		
Saline	3/3/3	3/3/3	3/3/3
BPC 157 10 µg/kg i.g.	0/0/0 *	0/0/0 *	0/0/0 *
BPC 157 10 ng/kg i.g.	0/0/0 *	0/0/0 *	0/0/0 *

**Table 2:** Lesions, scored microscopically, cerebrum, cerebellum, hypothalamus, and hippocampus in rats at 15 min, 5 h, or 24 h following unilateral adrenalectomy. Min/Med/Max, means ± SD, * *p* < 0.05, *at least*, vs. *control*. # combined score (0–8)–semiquantitative neuropathological scoring system; the sum of affected areas with infarction and karyopyknotic cells.

Medication	Lesions, Scored Microscopically Cerebrum, Cerebellum, Hypothalamus, and Hippocampus in Rats at 15 min (i), 5 h or 24 h min (ii, iii) Following Unilateral Adrenalectomy		
	15 min	5 h	24 h
	**Cerebrum (scored 0–8, Min/Med/Max) #**		
Control	2/3/3	2/3/3	3/3/3
BPC 157 10 μg/kg	0/1/1 *	0/1/1 *	0/1/1 *
BPC 157 10 ng/kg	0/1/1 *	0/1/1 *	0/1/1 *
	Neuronal damage in the karyopyknotic areas, %, means ± SD (10 HPF, 400×)		
Control	47 ± 5	63 ± 5	72 ± 5
BPC 157 10 μg/kg	10 ± 5 *	11 ± 5 *	9 ± 5 *
BPC 157 10 ng/kg	9 ± 5 *	12 ± 5 *	9 ± 5 *
	Hemorrhage (% of total area)		
Control	20 ± 3	30 ± 3	35 ± 3
BPC 157 10 μg/kg	0 ± 0 *	0 ± 0 *	0 ± 0 *
BPC 157 10 ng/kg	0 ± 0 *	0 ± 0 *	0 ± 0 *
	Edema (scored 0–3, Min/Med/Max)		
Control	2/3/3	3/3/3	3/3/3
BPC 157 10 μg/kg	0/1/1 *	0/1/1 *	0/1/1 *
BPC 157 10 ng/kg	0/1/1 *	0/1/1 *	0/1/1 *
	**Cerebellum (scored 0–8, Min/Med/Max)**		
Control	2/2/2	2/2/2	3/3/3
BPC 157 10 μg/kg	0/1/1 *	0/1/1 *	0/1/1 *
BPC 157 10 ng/kg	0/1/1 *	0/1/1 *	0/1/1 *
	Neuronal damage in the karyopyknotic areas, %, means ± SD (10 HPF, 400×)		
Control	56 ± 5	59 ± 5	74 ± 5
BPC 157 10 μg/kg	8 ± 5*	13 ± 5*	11 ± 5*
BPC 157 10 ng/kg	9 ± 5*	12 ± 5*	12 ± 5*
	Hemorrhage (% of total area)		
Control	0 ± 0	0 ± 0	0 ± 0
BPC 157 10 μg/kg	0 ± 0	0 ± 0	0 ± 0
BPC 157 10 ng/kg	0 ± 0	0 ± 0	0 ± 0
	Edema (scored 0–3, Min/Med/Max)		
Control	2/3/3	2/3/3	3/3/3
BPC 157 10 μg/kg	0/1/1 *	0/1/1 *	0/1/1 *
BPC 157 10 ng/kg	0/1/1 *	0/1/1 *	0/1/1 *
	**Hippocampus (scored 0–8, Min/Med/Max)**		
Control	2/3/3	2/3/3	2/3/3
BPC 157 10 μg/kg	0/1/1 *	0/1/1 *	0/1/1 *
BPC 157 10 ng/kg	0/1/1 *	0/1/1 *	0/1/1 *
	Neuronal damage in the karyopyknotic areas, %, means ± SD (10 HPF, 400×)		
Control	45 ± 5	51 ± 5	67 ± 5
BPC 157 10 μg/kg	2 ± 1 *	4 ± 1 *	12 ± 2 *
BPC 157 10 ng/kg	3 ± 1 *	3 ± 1 *	14 ± 2 *
	Hemorrhage (% of total area)		
Control	0 ± 0	0 ± 0	0 ± 0
BPC 157 10 μg/kg	0 ± 0	0 ± 0	0 ± 0
BPC 157 10 ng/kg	0 ± 0	0 ± 0	0 ± 0
	Edema (scored 0–3, Min/Med/Max)		
Control	2/2/3	2/2/3	2/3/3
BPC 157 10 μg/kg	0/0/0 *	0/0/0 *	0/0/0 *
BPC 157 10 ng/kg	0/0/0 *	0/0/0 *	0/0/0 *
	**Hypothalamus (scored 0–8, Min/Med/Max)**		
Control	2/3/3	2/3/3	2/3/3
BPC 157 10 μg/kg	0/1/1 *	0/1/1 *	0/1/1 *
BPC 157 10 ng/kg	0/1/1 *	0/1/1 *	0/1/1 *
	Neuronal damage in the karyopyknotic areas, %, means ± SD (10 HPF, 400×)		
Control	68 ± 5	72 ± 5	81 ± 5
BPC 157 10 μg/kg	21 ± 2 *	32 ± 2 *	43 ± 3 *
BPC 157 10 ng/kg	24 ± 2 *	35 ± 2 *	40 ± 3 *
	Hemorrhage (% of total area)		
Control	0 ± 0	0 ± 0	0 ± 0
BPC 157 10 μg/kg	0 ± 0 *	0 ± 0 *	0 ± 0 *
BPC 157 10 ng/kg	0 ± 0 *	0 ± 0 *	0 ± 0 *
	Edema (scored 0–3, Min/Med/Max)		
Control	2/3/3	3/3/3	3/3/3
BPC 157 10 μg/kg	0/1/1 *	0/1/1 *	0/1/1 *
BPC 157 10 ng/kg	0/1/1 *	0/1/1 *	0/1/1 *

**Table 3:** Summary of statistical comparisons across systemic, organ-specific, functional, morphometric, and molecular endpoints (*n* = 6 per group).

Domain	Outcome Type	Time Points	Test	Mean Difference Range	95% CI	*p*-Value	Effect Size
**Peripheral** **organs**	Ordinal lesion scores (0–3; 0–15)	15 min, 5 h, 24 h	Welch t (approx.)	−1 to −5	Non-overlapping/narrow	<0.0001	Very large *
**Peripheral** **organs**	Continuous lesion size/morphology	15 min, 5 h, 24 h	Welch t	−2 to −77	Non-overlapping	10^−10^–10^−12^	d = 10–22
**Heart function**	Heart rate (beats/min)	15 min, 5 h, 24 h	Welch t	−60 to −90	Narrow	<0.0001	d = 5–10
**Heart function**	QTc interval (ms)	15 min, 5 h, 24 h	Welch t	+30 to +60	Narrow	<0.0001	d = 5–9
**Hemodynamic system**	Blood pressure (SSS, portal, caval, aorta)	15 min, 5 h, 24 h	Welch t	normalization range	Non-overlapping	10^−6^–10^−12^	Very large
**Endocrine** **system**	Cortisol levels	15 min, 5 h, 24 h	Welch t	−40% to −80%	Narrow	10^−6^–10^−12^	Very large
**Adrenal gland**	Weight/ morphology	All	Welch t	+15% to +40% preservation	Non-overlapping	<0.0001	Very large
**Adrenal cortex**	Zona glomerulosa damage (%)	All	Welch t	−50% to −80%	Narrow	<0.0001	Very large
**Adrenal cortex**	Zona fasciculate damage (%)	All	Welch t	−40% to −75%	Narrow	<0.0001	Very large
**Brain**	Ordinal lesion scores (0–8; 0–3)	All	Welch t	−2 to −3	Complete separation	<0.0001	Very large *
**Brain**	Neuronal damage (%)	All	Welch t	−37% to −63%	Non-overlapping	<10^−10^	d = 7–13
**Brain**	Hemorrhage (%)	All	Welch t	−20% to −35/0	Non-overlapping or null	<0.0001/n.s.	Very large/0
**Brain**	Edema (0–3)	All	Welch t	−2 to −3	Complete separation	<0.0001	Very large *
**Vascular system**	Thrombosis	All	Fisher/Welch	−100% (abolished)	Complete separation	<0.0001	Maximal
**Systemic** **morphometry**	Integrated volume collapse/ reversal (brain, heart, vessels)	15 min, 5 h, 24 h	Welch t (derived ratios)	−40% to +90% reversal	Non- overlapping	10^−6^–10^−12^	Very large
**Redox status**	MDA (nmol/mg protein)	15 min, 5 h, 24 h	Welch t	normalization vs. healthy/ suppression vs. control	Narrow	<0.0001	Very large
**Nitrosative** **signaling**	NO levels (nmol/mg protein)	15 min, 5 h, 24 h	Welch t	increased vs. control	Narrow	<0.0001	Very large
**Gene expression (adrenal)**	NOS1, NOS2, NOS3, VEGFA (2^−ΔΔCt^)	15 min, 5 h, 24 h	Welch t	upregulation in treated groups	Narrow	<0.0001	Very large

* Very large = complete or near-complete group separation (variance ≈ 0; Cohen’s d becomes unstable or maximal).

**Table 4:** Specifications for TaqMan Assays genes and primers used in the experiment (Rat Genome Database and Alliance of Genome Resources nomenclature data).

Gene Symbol	Synonyms	Gene Name	TaqMan Assay ID	NCBI Reference Sequence	Amplicon Length (bp)
Gapdh	Gapd	Glyceraldehyde-3-phosphate dehydrogenase	Rn01775763_g1	NM_017008.4	174
Nos1	nNOS, bNOS	Nitric oxide synthase 1	Rn00583793_m1	NM_052799.1	65
Nos2	iNos, Nos2a	Nitric oxide synthase 2	Rn00561646_m1	NM_012611.3	77
Nos3	eNos, cNOS	Nitric oxide synthase 3	Rn02132634_s1	NM_021838.2	117
Vegfa	VEGF-A, VPF	Vascular endothelial growth factor A	Rn01511601_m1	NM_001110333.2	69

## Data Availability

The original contributions presented in the study are included in the article. Further inquiries can be directed to the corresponding authors.
